# Learning the Path to Ion Separation: A Proof-of-Concept for Machine Learning-Assisted Inverse Design (MLAID) of Nanofiltration Membranes

**DOI:** 10.1021/acs.est.5c15241

**Published:** 2026-09-29

**Authors:** Nohyeong Jeong, Elif Demirel, Changyoon Jun, Yongsheng Chen

**Affiliations:** School of Civil & Environmental Engineering, 1372 Georgia Institute of Technology, Atlanta, Georgia 30332, United States

**Keywords:** nanofiltration, reverse osmosis, machine learning, explainable artificial intelligence, membrane separation mechanisms, inverse design

## Abstract

Advancing the design of polymeric membranes through material screening and discovery offers a powerful strategy to guide future membrane fabrication tailored to a wide range of separation challenges. Traditional trial-and-error methods often fall short in navigating this large monomer and process-design space, whereas machine learning-assisted inverse design (MLAID) can provide an extensible workflow for translating literature-derived membrane knowledge into ranked experimental candidates. Building on prior inverse-design strategies that combine machine learning (ML) with Bayesian optimization (BO), this study applies MLAID to prioritize membrane candidates within a target-defined nanofiltration (NF) design space. MLAID models are trained with membrane fabrication data, and their underlying patterns are interpreted through Shapley additive explanations (SHAP). SHAP was used to investigate model-attribution patterns, and BO was used to prioritize monomer and fabrication-condition combinations for producing polyamide membranes with targeted permeability and selectivity. The MLAID framework prioritized candidate formulations across target salt-water permeabilities of 4 to 12 LMH/bar (L m^–2^ h^–1^ bar^–1^), with Na_2_SO_4_ rejection used as the experimental benchmark. Fabrication and testing served as the experimental decision gate for evaluating the model-prioritized candidates. Overall, this study demonstrates how ML/BO-guided prioritization can accelerate identification of promising monomers and fabrication conditions within an encoded design space, while providing a workflow backbone that can expand as richer target-specific data sets, descriptors, and validation become available.

## Graphical abstract

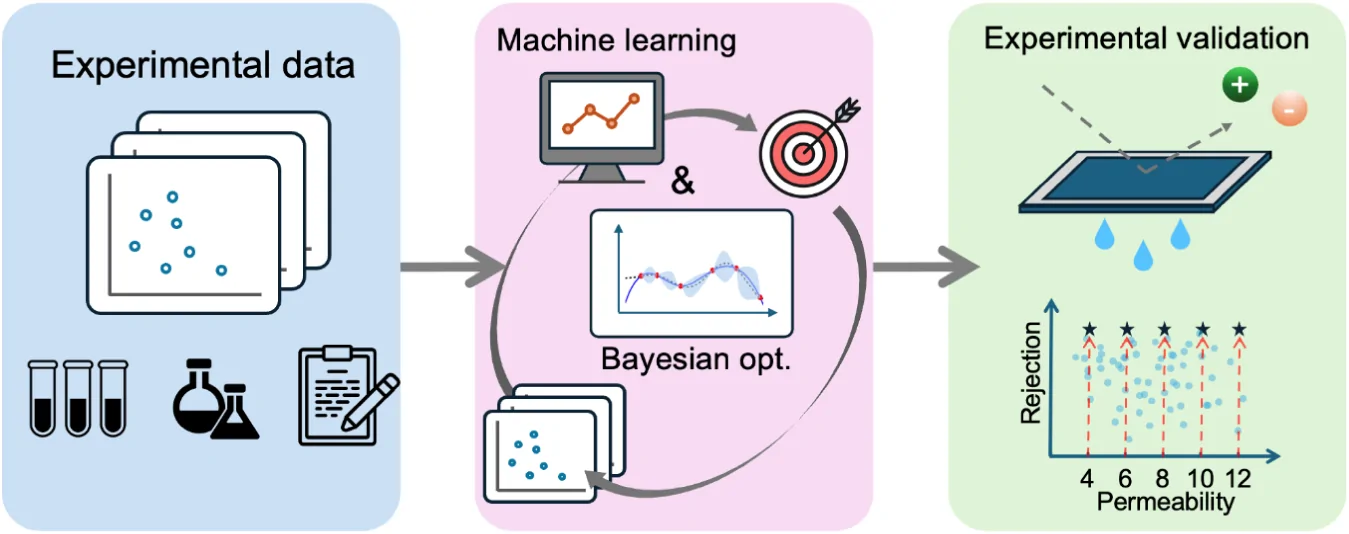

## Introduction

Global water security is increasingly threatened by growing water scarcity and the widespread contamination of resources by a diverse array of pollutants, including microorganisms, heavy metals, pharmaceuticals, microplastics, personal care products, and persistent organic pollutants such as pesticides and per- and polyfluoroalkyl substances (PFAS).^1–4^ Nanofiltration (NF) membranes offer a promising solution for both pollutant removal and efficient resource recovery, including water, nutrients, salts, minerals, solvents, and valuable industrial chemicals, from diverse water matrices. Their high permeability, selective separation capabilities, low energy requirements, and broad applicability make them ideal for sustainable treatment processes.^5–7^ Interfacial polymerization (IP) is the most widely used method for fabricating NF membranes, enabling the formation of an ultrathin selective layer essential for high separation performance. However, the process is complex and governed by numerous factors, including monomer type and composition, polymerization time, curing conditions, and the properties of the support material, each of which can significantly influence membrane structure and separation efficiency.^8,9^

The impacts of fabrication conditions on membrane morphology and performance have been extensively investigated, providing valuable insights into the structure–property-performance relationships.^10,11^ One of the key challenges in membrane science is optimizing fabrication parameters to overcome the inherent trade-off between permeability and selectivity for targeted separation tasks.^12,13^ Accordingly, recent research has focused on overcoming this trade-off by developing novel strategies to enhance water permeability without compromising solute rejection, or vice versa, paving the way for more efficient and targeted membrane separations.^14,15^ In particular, recent research has focused on strategies such as incorporating nanoparticles, including graphene oxide (GO),^16,17^ MXenes,^18^ carbon nanotubes (CNTs),^19^ metal–organic frameworks (MOFs),^20^ quantum dots^21^ and their blends,^22^ as interlayers or additives to regulate IP. Additionally, alternative monomers,^23,24^ surface modifications,^25^ and post treatment^26^ methods have been explored to tailor membrane properties for improved performance.

Although experiment-driven approaches, including those guided by design of experiments (DoE), have led to notable progress, they remain constrained by several limitations.^27,28^ Membrane fabrication is often complex, resource-intensive, and costly, posing challenges for large-scale implementation. Reproducibility and consistency across batches are frequently problematic, and performance gains tend to only marginally alleviate the inherent trade-off between permeability and solute rejection. Additionally, traditional methods rely heavily on researchers’ intuition and experience, which can lead to unpredictable outcomes, especially when the structure-performance relationship is not fully understood.

To overcome these limitations, recent research has increasingly embraced data-driven and artificial intelligence (AI)-guided approaches that reverse the conventional trial-and-error process. These methods enable more efficient exploration of material design space and accelerate performance optimization, paving the way for smarter and more scalable membrane fabrication.^29^ Machine learning (ML) serves as a powerful tool for capturing complex, nonlinear relationships between system variables by identifying patterns within large data sets. When implemented through advanced algorithms and trained on experimental or simulated data sets, ML can significantly accelerate the discovery of high-performance membrane formulations. It also facilitates process optimization and performance evaluation, ultimately guiding the rational development of next-generation membranes with enhanced separation capabilities.^30^ To date, ML has been widely applied in membrane science for various purposes such as property prediction,^31–33^ interpretation of membrane features, and performance characteristics,^34–36^ polymer screening, and optimization of design parameters.^37–39^ However, its application in the discovery of new materials through inverse membrane design remains largely underexplored. Recent studies have addressed this gap by directly linking monomer chemistry to membrane performance using data-driven approaches.^44^ For example, fragment-based ML models that decomposed monomers into chemically meaningful substructures (e.g., functional groups and molecular fragments) were developed to quantitatively relate monomer composition to membrane permeance and solute rejection. These models, trained on integrated data sets of monomer structure, fabrication conditions, and membrane performance, achieved high predictive accuracy by enabling the identification of key chemical motifs governing separation behavior.^40^

In parallel, recent developments in explainable artificial intelligence (XAI) provided deeper mechanistic insights into membrane separation processes. Approaches based on Shapley additive explanations (SHAP) analysis and causal learning enabled the decoupling of complex interactions such as steric hindrance, electrostatic effects, and solute-membrane affinity, offering a more physically meaningful interpretation of separation mechanisms. These approaches provide transparency by quantifying the contribution of individual features and revealing their influence on model outputs, thereby bridging the gap between data-driven predictions and physical understanding of how input features govern membrane performance.^41,42^ In this context, recent studies have emphasized that the reliability and generalizability of ML models in membrane science are strongly dependent on adherence to best practices, including high-quality and sufficiently diverse data sets, appropriate feature engineering, rigorous validation strategies, and transparent model reporting. These guidelines highlight that model performance is inherently limited by data availability and distribution, and that imbalanced or sparse data sets can lead to reduced predictive accuracy, particularly when extrapolating beyond the training domain.^43^ Furthermore, recent large-scale data-driven studies have expanded the scope of ML beyond performance prediction toward understanding membrane formation mechanisms. The development of extensive IP data sets has enabled the prediction of thin-film formation directly from monomer structures, marking a significant step toward high-throughput and predictive membrane fabrication. In particular, the inclusion of both successful and unsuccessful reactions has facilitated the development of classification models that capture the conditions governing film formation, highlighting the importance of negative data and broad chemical space coverage in data-driven membrane design.^44^

Building on these advances, AI has facilitated groundbreaking advances in materials discovery, significantly accelerating every phase of the process and expanding design space by leveraging large data sets.^45^ The utilization of advanced ML algorithms has enabled accurate predictions of material properties, structural and functional modifications of high potential candidates from an infinite design space by effectively analyzing extensive complex data patterns.^46^ While material discovery and design of membranes are relatively new research areas, studies in this domain remain limited. Most of the ML studies in membrane science have focused on predicting, analyzing, and optimizing process conditions across various membrane technologies.^47^ Within this context, inverse membrane design has emerged as a transformative strategy. In inverse membrane design, desired performance targets such as permeability, rejection or selectivity are specified first, followed by identification of optimal monomer chemistries and fabrication conditions.^30,37^

By leveraging Bayesian optimization (BO) and model interpretation tools like SHAP, ML models can reveal how structural features influence membrane performance. This ML-driven framework enables efficient virtual screening and optimization of membrane fabrication conditions, while significantly reducing the time, cost, and inefficiency associated with traditional methods.^37^ Considering that inverse membrane design is still at an early stage of development, there remains a need to extend such workflows toward target-defined, experimentally actionable membrane formulations across diverse applications.

Prior ML/BO-guided polyamide (PA)-NF membrane-design work demonstrated that ML models can be coupled with BO to identify promising monomer and fabrication-condition combinations and experimentally test selected candidates near the permeability-selectivity trade-off.^39^ Building on this direct foundation and the broader workflow logic summarized in recent reviews,^37^ this study presents an ML-guided inverse design framework for NF membranes across a broader range of target salt-water permeabilities. The selected formulations were evaluated using Na_2_SO_4_ rejection as the experimental benchmark under defined testing conditions. In addition, we refined the input feature set by excluding features that were insufficiently represented in the data set, thereby reducing unnecessary model complexity while preserving meaningful structure–property relationships. We also adopted salt-water permeability as the inverse design target to more directly capture the permeability-selectivity trade-off relevant to NF applications.

Leveraging a curated literature-derived data set, we trained predictive models and applied BO alongside SHAP to systematically screen monomers and process parameters. This approach enabled the prioritization of membrane candidates targeting water permeabilities ranging from 4 to 12 LMH/bar while maintaining high salt rejection as a design criterion. Experimental fabrication and testing evaluated the selected formulations. The membranes were further characterized using SEM imaging, zeta potential measurements, MWCO analysis, and particle size distribution. These analyses provide physical context for the observed transport behavior of the selected membranes. Together, these steps position MLAID as a target-defined workflow for translating literature-derived evidence into experimentally evaluated NF candidates and for identifying design-space boundaries that can guide future descriptor and data set development.

## Materials and Methods

### Materials

1,3-cyclohexanebis­(methylamine) (98%), m-phenylenediamine (99%), o-phenylenediamine (99.5%), piperazine (99%), trans-2,5-dimethylpiperazine (98%), 2,5-piperazinedione, triethylamine (99.5%) and 1,3,5-benzenetricarbonyl trichloride (TMC; 98%) were purchased from Sigma-Aldrich. Anhydrous *n*-hexane and Na_2_SO_4_ (analytically pure) were supplied by Thermo Scientific Chemicals and Research Products International, respectively. A commercial poly­(ether sulfone) (PES) membrane (0.45 μm pore size) (Sterlitech, Auburn, WA, USA) was used as the substrate for the fabrication of the polyamide layers. Ultrapure water (18.25 MΩ·cm) was used for all the experiments and all chemicals were used as received without further purification.

### Membrane Fabrication

Polyamide membranes were fabricated using IP to validate the results of the ML-assisted inverse design framework. The detailed fabrication procedure was described in our previous study.^39,48^ Briefly, an aqueous solution containing selected monomers and an additive was prepared, along with an organic solution of TMC in *n*-hexane. The concentrations of monomers and additives were determined by the ML-assisted inverse design framework. While *n*-hexane and TMC were fixed as organic solvent and organic phase monomer, respectively, the aqueous phase monomers and additives varied depending on the optimization results from the inverse design framework. A PES microfiltration support membrane (0.45 μm) was placed on a clean glass plate and secured with a funnel. The aqueous solution was poured onto the surface to impregnate it for 3 min, followed by draining the aqueous solution and air drying the membrane for several minutes. Subsequently, the organic solution was applied to the membrane surface to initiate IP. After a certain polymerization time, the excess solution was discarded, and the membrane was thermally cured under specified temperature and time. The resulting membranes were stored in Milli-Q water until further testing. The fabrication parameters, including specific monomer types, additive concentrations, polymerization time, and curing conditions are summarized in Table 1.

**Table 1:**
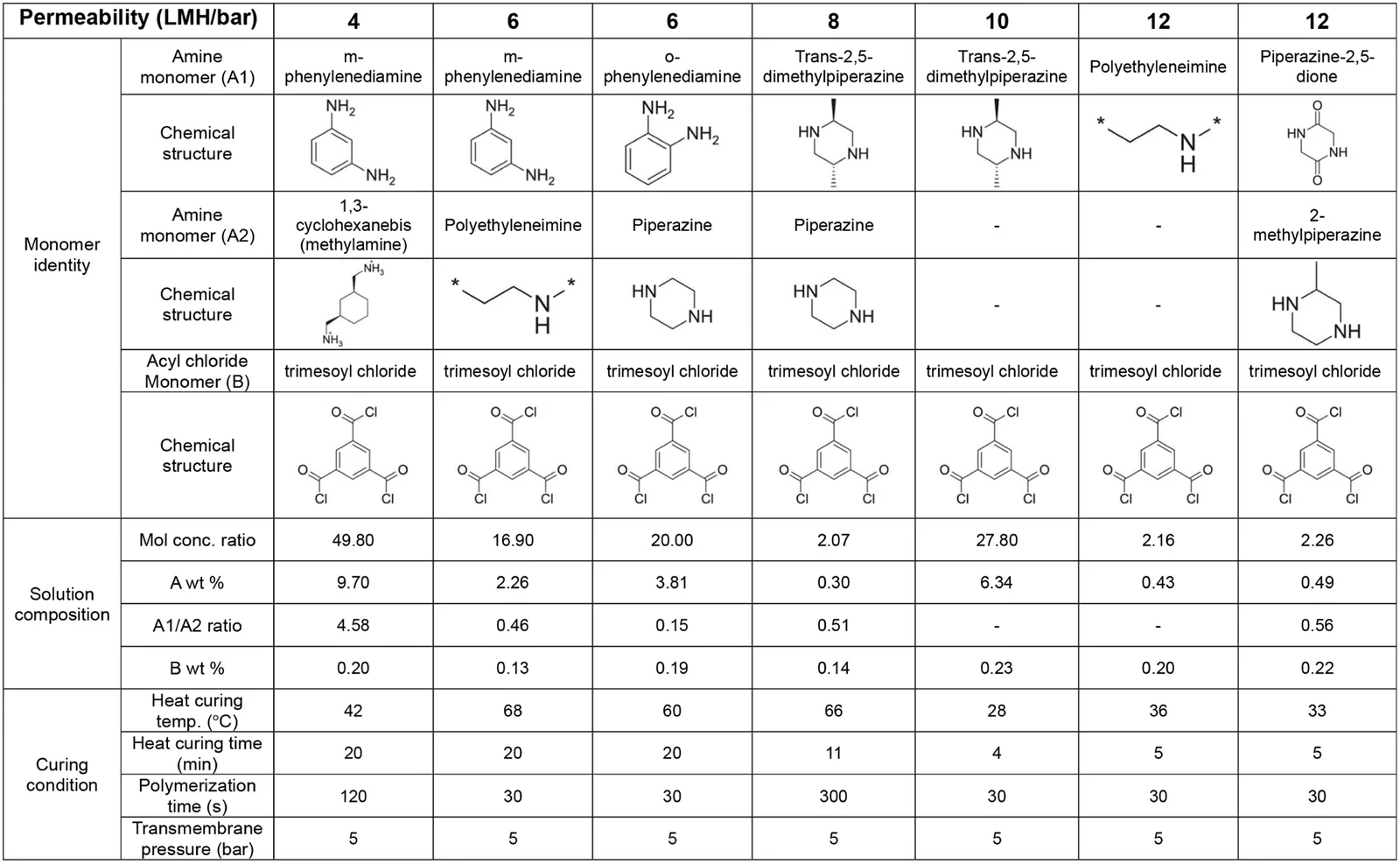
Optimized Membrane Fabrication Conditions for Targeted Water Permeability (4, 6, 8, 10, and 12 LMH/bar) with High Salt Rejection

### Membrane Performance Test

Membrane performance tests were conducted using a lab-scale cross-flow filtration system with an effective membrane area of 4.1 cm^2^. Prior to each test, membranes were compacted with Milli-Q water at 7 bar to stabilize flux. All experiments were performed at a constant transmembrane pressure of 5 bar and a temperature of 20 °C. A cross-flow velocity of 0.5 m/s was maintained throughout the tests. Following each water permeability test, a 1,000 mg/L Na_2_SO_4_ solution was used as the feed to evaluate salt rejection. The electrical conductivities of both the feed and permeate were measured using a conductivity meter, and salt rejection was calculated accordingly. The mass of the permeate was monitored at 1 min intervals using a PS 8100.X2.M Precision Balance (Radwag, Poland) connected to data acquisition software. Each measurement was performed in triplicate using membranes fabricated through three independent IP processes. The water permeability coefficient (A), salt rejection (R), solute permeability coefficient (B), and water/salt selectivity (A/B) were calculated using the following equations.^49^

1 $$A=\frac{J_{w}}{\left(\Delta P-\Delta \pi \right)}$$

2 $$R=1-\frac{C_{p}}{C_{f}}=\frac{J_{w}}{J_{w}+B}$$

3 $$B=\frac{\left(1-R\right)J_{w}}{R}$$

4 $$\frac{A}{B}=\frac{R}{\left(1-R\right)\left(\Delta P-\Delta \pi \right)}$$

where Δ*P* is the applied pressure, Δ*π* is the osmotic pressure difference across the membrane, *C_f_* and *C_p_* are concentrations of salt in feed and permeate solutions.

### Membrane Characterization

Membranes were characterized to elucidate structural and surface properties and to provide physical context for the observed transport behavior. Surface and cross-sectional morphologies were examined using field emission scanning electron microscopy (FESEM, Hitachi S-4700, Tokyo, Japan). Membrane samples were cut into small sections, dried, and mounted on aluminum stubs using conductive carbon tape followed by sputter-coating with a thin gold–palladium layer to minimize charging. For cross-sectional analysis, membranes were freeze-fractured in liquid nitrogen and mounted vertically prior to coating. Surface zeta potential was measured using a Malvern Zetasizer Nano ZS (Malvern, UK) with a surface zeta potential cell. Membrane samples were mounted in the cell and immersed in 1 mM KCl solution at pH 7. Measurements were conducted in triplicate and averaged. Molecular weight cutoff (MWCO) was determined based on the retention toward a series of neutral organic solutes (glycerol and polyethylene glycol (PEG) with different molecular weights). The MWCO was defined as the molecular weight corresponding to 90% solute rejection, while the mean pore size was taken as the Stokes radius of the solute with 50% rejection. The Stokes radii of neutral organic molecules (r_p_) are correlated with their molecular weight (MW).

For glycerol (92 Da):

5 $$\log r_{p}=-1.4962+0.4654\log\left(MW\right)$$

For PEGs (MW of 200, 300, 400, and 600 Da):

6 $$r_{p}=16.73\times {\left(MW\right)}^{0.557}\times 10^{-3}$$

The mean pore size was calculated as a log-normal probability density function:

7 $$\frac{dR\left(r_{p}\right)}{dr_{p}}=\frac{1}{r_{p}\ln\sigma_{p}\sqrt{2\pi }}\exp\left(-\frac{{\left(\ln r_{p}-\ln\mathrm{r̅}\right)}^{2}}{2{\left(\ln\sigma_{p}\right)}^{2}}\right)$$

where *σ_p_* and r̅ denote the geometric standard deviation and mean pore size of a membrane, respectively.^50^

### Data Collection and Variable Selection

To ensure effective ML training, input variables were carefully selected based on the key factors influencing membrane fabrication. Numerical variables were chosen to capture distinct and complementary aspects of fabrication, and pairwise correlations among key variables were examined to minimize detrimental redundancy while preserving mechanistically meaningful information (Figure S1, Supporting Information). These variables were categorized into three groups: categorical features, numeric features, and chemical structures, the latter represented by Morgan fingerprints. For categorical features, we included types of organic solvents and additives used in the aqueous solution. The numeric features comprised the concentration ratio of aqueous-phase monomer A1 to A2 (A1/A2 ratio), transmembrane pressure, polymerization time, heat curing time and temperature, as well as the concentrations of additives in the aqueous solution. Additionally, we considered the physicochemical properties of cations and anions such as valence, ionic radius, hydrated radius, and hydration energy, and types of monomers used in both the aqueous and organic phases. In addition to the input variables used in the previous study,^39^ we incorporated the mole ratio between monomer A (i.e., amine monomer) in the aqueous solution and monomer B (i.e., acyl chloride) in the organic solution, along with the sum of their weight percentages. Including this stoichiometric information enables the ML models to better capture the influence of chemical reaction conditions on membrane performance. Chemical structural information was represented using Morgan fingerprints. Morgan fingerprints with a length of 2048 bits and a radius of 0 were used, where each bit indicates activation of a hashed local atomic-environment descriptor. The fingerprint therefore captures structural similarity through the collective pattern of activated bits rather than through explicit functional-group assignments.

A complete list of input variables, including their full names and abbreviations, is provided in Table S1 (Supporting Information). The distributions of permeability and salt rejection values, as well as the chord diagram showing the combinations of monomers in data sets, are illustrated in Figures S2–S4 (Supporting Information). Extreme fabrication conditions (heat curing time > 200 min) and samples lacking simplified molecular-input line-entry system (SMILES) representations for the monomers during data preprocessing were excluded from the data set, improving data set consistency. Specifically, 12 samples were removed due to excessive heat-curing time, and an additional 84 samples were excluded due to unavailable monomer SMILES representations. ML models were built to predict water permeability and salt rejection using model-specific input-variable sets detailed in Table S1. Model training was performed using stratified 5-fold cross-validation, and Bayesian optimization was utilized to fine-tune the hyperparameters. In this study, the permeability indicates salt-water permeability rather than pure-water permeability. Within this regime, membrane samples exhibiting salt-water permeability above 10 LMH/bar while maintaining meaningful solute rejection are intrinsically rare in both experimental studies and literature. As a result, the available data set is naturally imbalanced toward lower and intermediate permeability values. This imbalance reflects not only experimental sampling limitations but also the practical and physical limitations of current NF and reverse osmosis (RO) membranes.

### ML Model and Interpretation Using SHAP

We employed extreme gradient boosting (XGBoost) to predict membrane performance, given its strong predictive capabilities in previous studies,^33,34,36,51,52^ and its robustness in handling missing input values. Bayesian optimization was used to tune the hyperparameters, and their ranges are presented in Table S2 (Supporting Information). During model training, a standard error-based loss function was used to minimize the discrepancy between predicted and measured values for each target variable independently, supporting stable learning of input-output relationships. Customized objective functions were applied only at the inverse-design stage, where the trained model outputs were combined using task-specific formulations to reflect distinct design priorities for salt rejection and water permeability.

SHAP analysis was employed to quantify and interpret the contribution of each input variable. Based on coalitional game theory,^53^ Lundberg and Lee proposed a practical method for attributing contributions to individual players.^54,55^ In this case, the players are the input variables, whose contributions are evaluated based on their marginal impacts on model predictions. SHAP values provide a quantitative measure of each variable’s contribution by comparing model predictions across feature coalitions with and without the variable of interest. The weighted average of these marginal contributions is defined as the SHAP value. The SHAP value for input variable *i*, denoted as Φ*_i_* (*p*), is derived from the model prediction p and can be calculated by the following equation.

8 $$\Phi_{i}\left(p\right)=\underset{S\subseteq D\backslash \left\{i\right\}}{\sum }\frac{\left|S\right|!\left(\left|D\right|-\left|S\right|-1\right)!}{\left|D\right|!}\left(p\left(S\cup \left\{i\right\}\right)-p\left(S\right)\right)$$

where *D* denotes the set of players (i.e., input variables), *D* = {1,...,*d*}, and *S* is a subset of *D* that excludes variable *i*. *p*(*S* ∪ {*i*}) denotes the model prediction when variable *i* is included, whereas p­(S) denotes the prediction without variable *i*. $\frac{\left|S\right|!\left(\left|D\right|-\left|S\right|-1\right)!}{\left|D\right|!}$ and *p*(*S* ∪ {*i*}) – *p*(*S*) represent the weight assigned to *S* and the marginal contribution of *i*, respectively.^53–55^ Using SHAP values derived from the Morgan fingerprints of monomers, we evaluated how encoded descriptor patterns were associated with salt rejection and water permeability predictions. Because Morgan fingerprints are hashed descriptors, these results are interpreted as model diagnostics rather than direct mechanistic assignments.

### Inverse Membrane Design Using ML-BO Framework

BO is a sequential, model-based approach for identifying the global maximum or minimum of an unknown objective function. It improves optimization efficiency by leveraging information from prior observations to update a posterior distribution over the objective function. This strategy consists of two key components: 1) a probabilistic surrogate model that estimates the target outcomes of the unknown objective function, and 2) an acquisition function that guides the sequential selection of new sampling points to maximize expected improvements. During iteration, the probabilistic surrogate model is updated with new observations to refine the posterior distribution over the unknown function.^56^ The next sampling point is determined by identifying the point that maximizes the acquisition function. Previous studies have demonstrated the effectiveness of BO in exploring complex design spaces.^57–60^

In this study, BO was used both to tune hyperparameters of ML models and to search membrane fabrication conditions based on the predictions of two performance metrics: salt rejection and water permeability. Hyperparameter tuning was conducted over 10,000 iterations, with the list of optimized parameters provided in Table S2 (Supporting Information). After training the XGBoost models to predict salt rejection and water permeability, we defined a loss function for the surrogate model used in ML-Bayesian optimization. This function guided the search for fabrication conditions that prioritize targeted performance outcomes, as described below. The chosen loss function was designed to reflect two equally essential and independent design requirements: 1) achieving high permeability and 2) maintaining high salt rejection. The two terms were therefore combined without additional weighting to avoid introducing application-specific bias during optimization. This formulation is intended as a practical screening objective rather than a unique global optimum, and alternative scalarizations or fully multiobjective formulations may yield different candidate sets.

9 $$\mathrm{Loss}\;\mathrm{function}=\frac{B}{A}+\left|permeability\;prediction-target\;permeability\right|$$

10 $$\frac{B}{A}=\frac{100-rej.pred.}{rej.pred.}\times \Delta P$$

where $\frac{B}{A}$ is the reciprocal of water/salt selectivity and Δ*P* represents the transmembrane pressure. For the optimization of membrane fabrication conditions, certain parameters were held constant for candidate evaluation based on their widespread use in the literature, specifically, the organic phase monomer (TMC), organic solvent (hexane), and transmembrane pressure (5 bar). The remaining fabrication conditions were optimized using an ML-Bayesian optimization approach. As shown in Figure 1, the ML models for salt rejection and water permeability were used to minimize two objectives: water/salt permeability ratio $\left(\frac{B}{A}\right)$ , and the absolute difference between the predicted and target water permeability values, as defined in eq 9.

**Figure 1:**
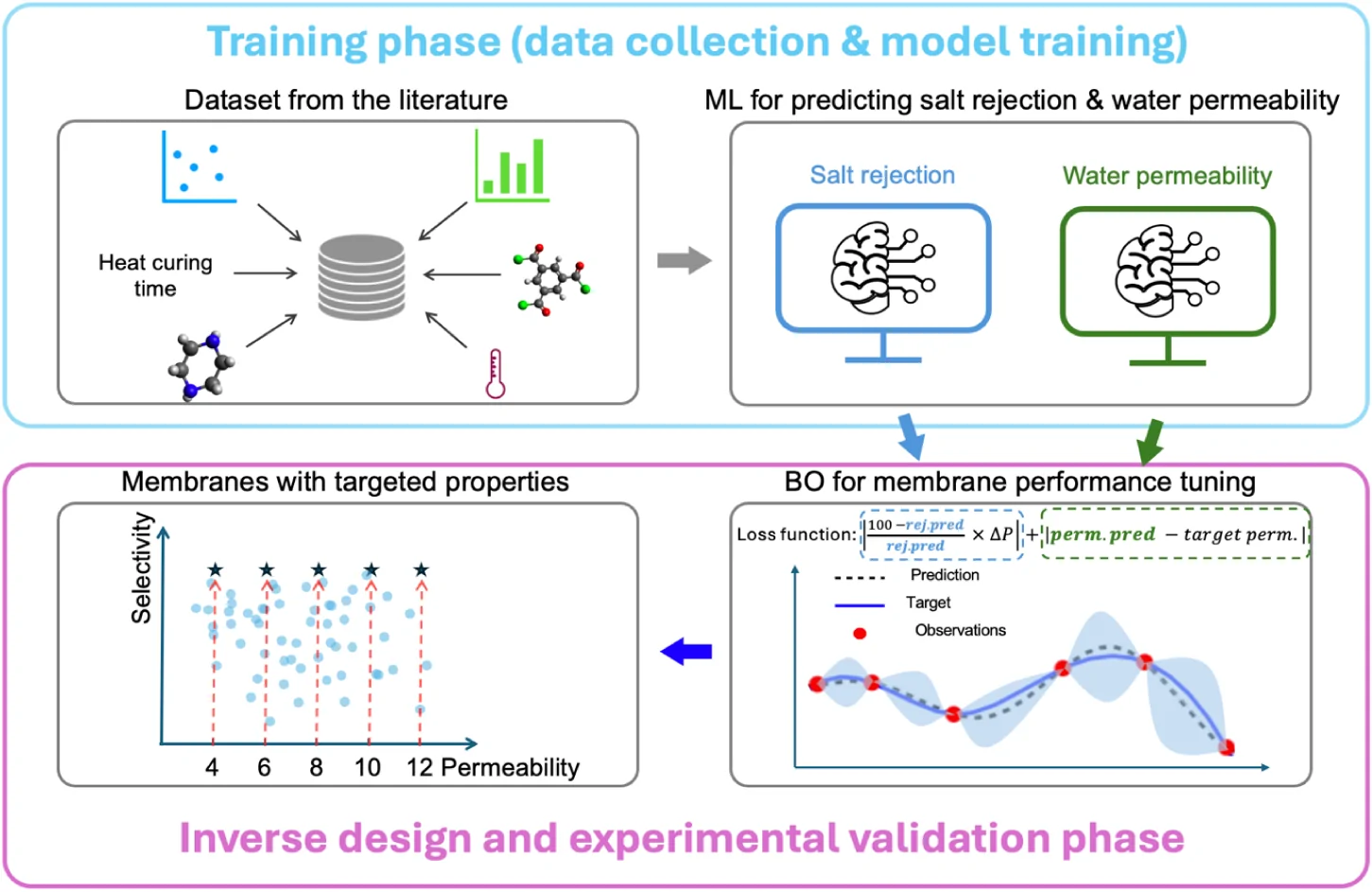
Schematic of the workflow for the optimization of membrane properties using ML and Bayesian optimization.

The list of 310 candidate monomers was obtained from the National Institute for Materials Science (NIMS) materials database for membrane design. From the list, commercially available monomers were selected for further optimization. To identify promising candidates, candidate monomers were screened using similarity to reference structures derived from aggregated fingerprint patterns and then constrained to commercially available compounds compatible with the experimental workflow. This approach narrows the design space and supports candidate prioritization. The Bayesian optimization procedure for inverse design was conducted over 10,000 iterations to thoroughly explore the design space. Among the generated candidates, selected fabrication conditions were synthesized and tested experimentally. Using this inverse design strategy, we optimized the membrane fabrication conditions to achieve target water permeability values of 4, 6, 8, 10, and 12 LMH/bar, along with high salt rejection, to explore the high-permeability edge of the permeability-selectivity trade-off.

## Results and Discussion

### ML Performance for Water Permeability and Salt Rejection Predictions

A comprehensive data set was compiled from the literature to develop an ML model for membrane performance prediction. As shown in Figure 1, we collected data on NF and RO membrane fabrication from studies published over the past 20 years, as detailed in our previous study.^39^ In that study, water permeability was calculated based on pure water flux. However, pure water flux does not accurately reflect the energy consumption of the membrane system (e.g., the effects of osmotic pressure, ion-membrane interactions, and concentration polarization) and can therefore overestimate effective system permeability, making it less representative of real-world applications. Thus, in this study, we utilized the permeate flux of salt solutions to calculate water permeability. As a result, the physicochemical properties of cations and anions in the salt solutions were incorporated into the ML training data.

The ML models for predicting salt rejection and water permeability were trained using 513 and 347 data points, respectively, representing 80% of the total data set, while the remaining 20% was held out for testing. During model training, 5-fold cross-validation was employed, with root mean squared error (RMSE) used as the evaluation metric. Model performance on the testing data was assessed using both RMSE and mean absolute error (MAE). The salt rejection model achieved an R^2^ of 0.87, an RMSE of 11.9%, and an MAE of 7.5%, while the water permeability model yielded an R^2^ of 0.63, an RMSE of 1.8 LMH/bar and an MAE of 1.2 LMH/bar (Figure 2). These model-performance results are consistent with previous ML studies on membrane performance prediction.^34,36,39^ For comparison, on the same held-out test splits used for Figure 2, XGBoost outperformed a training-set-mean baseline for both targets (Table S6).

**Figure 2:**
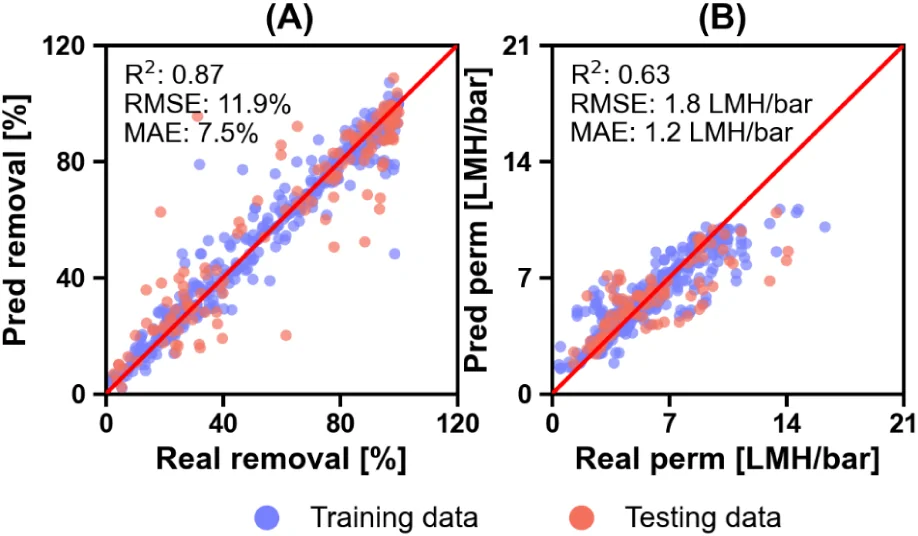
Prediction performance of ML models for (A) salt rejection and (B) water permeability.

In Figure 2A, the deviations in salt rejection prediction are distributed across the entire range of rejection values without a clear bias toward a certain range. However, larger errors were observed at higher permeability values, where data density is lower (Figure 2B). The observed discrepancies partly reflect the literature-derived nature of the data set: results compiled under nonstandardized conditions across laboratories introduce irreducible noise that the available input variables cannot fully capture. In addition, residual analyses were conducted to investigate model performance as a function of predicted values and key input variables. As shown in Figures S5–S7, prediction errors increase in data-sparse regimes, particularly at high permeability values and for monomers with limited representation in the data set. Additional repeated random-row split and same-input-condition exclusion analyses are provided in Tables S3–S5. Because random splitting may retain publication-specific patterns, these results support candidate prioritization within the assembled literature data set, while cross-study transfer remains to be evaluated with broader source coverage. The overall agreement between predicted and measured values in Figure 2 provides a practical basis for prioritizing fabrication conditions.

### ML Interpretation Using SHAP Method

Interpreting the decision-making process of ML models remains challenging due to their inherent “black box” nature. To effectively use ML for decision-making workflows, it is essential to build trust in the ML models. While enhancing predictive accuracy is often a primary goal in AI-driven environmental applications, strong performance alone does not guarantee that an ML model is reliable enough to support critical decisions.^61^ In addition to confidence in prediction accuracy, understanding the underlying reasoning and patterns captured by the model is equally important. Model interpretability ensures that predictions are grounded in meaningful domain knowledge rather than misleading correlations, thereby supporting transparent and justifiable ML-driven decisions.

To interpret ML models developed for predicting salt rejection and water permeability, we implemented the SHAP method. SHAP values quantify the contribution of each input variable to the model’s predictions. Figure 3 presents SHAP summary plots for both models, with variables ranked by their relative importance along the *y*-axis. Notably, the key influential variables differ between the salt rejection and water permeability models, highlighting the need to consider all relevant factors when designing membranes that perform well for both metrics. Figure 3A shows that anion valence (An_val), the molar ratio of monomer A to monomer B (Mol_ratio), cation valence (Cat_val), and monomer B concentration (B conc.) were the most influential variables in predicting salt rejection. SHAP dependence plots of input variables with high SHAP values for salt rejection and permeability models are provided in Figure S8 (Supporting Information). More negative anion-valence values, corresponding to multivalent anions such as SO_4_^2–^ (valence = −2), showed a positive contribution to salt-rejection predictions, whereas less negative values correspond to monovalent anions such as Cl^–^ (valence = −1). As shown in Figure S9A (Supporting Information), SHAP values for anion valence are positive when the anion valence exceeds that of the cation. Conversely, when cation valence is higher, the SHAP values for cation valence increase. Although electrostatic interactions are important in both scenarios, anion valence exerts a stronger influence on salt rejection than cation valence. This trend can be attributed to the negatively charged surface of PA-based NF and RO membranes, which typically exhibit a negative charge under standard experimental pH conditions.^62^ For asymmetric salts (e.g., Na_2_SO_4_, MgSO_4_, CaCl_2_, and MgCl_2_), ion rejection is primarily governed by the ion sharing the same charge sign as the membrane surface.^63,64^ Given the negatively charged nature of the membrane, anion valence plays a more prominent role in determining salt rejection performance.

**Figure 3:**
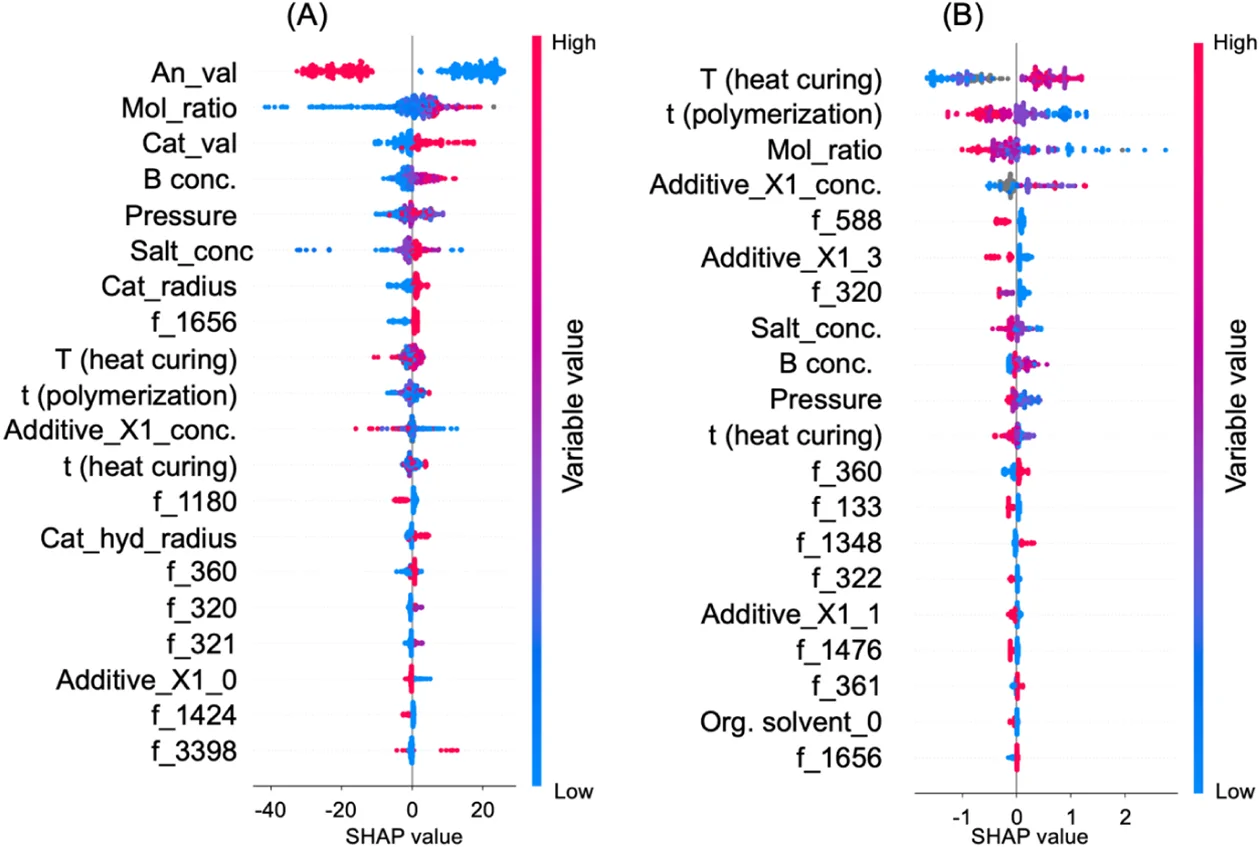
SHAP summary plots for ML models predicting (A) salt rejection and (B) water permeability. The full name and abbreviation of each input variable are listed in Table S1.

Other important variables influencing salt rejection include the molar concentration ratio (Mol_ratio) of the amine monomer (monomer A) to the acyl chloride monomer (monomer B), as well as the concentration of the acyl chloride monomer (B conc.). An increase in the molar concentration ratio corresponds to improved salt rejection, indicating that a relatively higher proportion of amine monomer enhances membrane solute rejection. During the fabrication of RO and NF membranes, polymerization mainly occurs on the organic side of the interface. To facilitate the diffusion of aqueous monomers and the formation of the separation layer, an excess concentration of aqueous monomers is often used. Insufficient monomer concentrations can result in a less cross-linked active layer or even a flawed separation layer with a poor selectivity.^65^ A similar trend is observed for the acyl chloride monomer, where higher concentrations are associated with increased SHAP values. These two variables jointly influence the degree of cross-linking between amine and acyl chloride monomers, leading to denser or looser structures depending on their ratio.^66^ This highlights the molar concentration ratio as a critical factor in predicting salt rejection. Although amine monomer concentration is important, it was not ranked highly in the SHAP summary plot. This is due to the model’s ability to infer its effect indirectly through the combined influence of the molar concentration ratio and acyl chloride monomer concentration.

The water permeability model is primarily influenced by heat curing time, polymerization time, molar concentration ratio of amine to acyl monomers (Mol_ratio), and the concentration of additive (Additive_X1_conc.). These variables are closely tied to the polymerization process; because water permeability is an intrinsic property of membrane structure, their strong influence on the model is expected. As shown in Figure 3B, higher heat curing temperatures appear to positively contribute to water permeability prediction. However, this trend is inconsistent with the experimental results reported in the literature. Micropores formed during interfacial polymerization often retain solvent, and subsequent heat curing facilitates condensation polymerization, leading to a higher degree of cross-linking and denser membrane pores.^67,68^ As a result, elevated heat-curing temperatures and longer heat-curing times typically reduce water permeability. The apparent discrepancy between the SHAP-based interpretation and experimental observations arises because heat-curing temperature does not behave as an independent variable in the literature-derived data set. Instead, heat curing temperature is frequently coadjusted with curing time and monomer concentration as part of coordinated experimental design choices. As illustrated in Figure S10 (Supporting Information), higher curing temperatures are often paired with shorter curing times, which can complicate marginal feature attribution. Consequently, the SHAP trend associated with curing temperature reflects correlations embedded in the data set rather than a direct causal physical relationship. Polymerization time and the molar concentration ratio significantly affect the density of cross-linking within the membrane matrix. Shorter polymerization time and lower molar concentration ratios typically result in reduced cross-linking. Notably, the molar concentration ratio exerts opposite effects on salt rejection and water permeability. As a key determinant of membrane cross-linking, this ratio highlights the inherent trade-off between permeability and selectivity, which is evident from the SHAP values associated with this variable in both models.

SHAP summary plots also reveal the impacts of encoded monomer structural descriptors, represented by Morgan fingerprint features (f_number), on both salt rejection and water permeability. Each monomer fingerprint is encoded as a binary vector, in which nonzero values indicate activation of a hashed local atomic-environment descriptor. The corresponding SHAP values indicate whether a given descriptor pattern is associated with higher or lower model predictions. It should be noted, however, that individual Morgan fingerprint bits do not correspond uniquely to specific functional groups or reaction motifs. Morgan fingerprints encode local atomic environments into a fixed-length hashed vector, and as a result, multiple distinct chemical environments may map to the same fingerprint index due to unavoidable bit collisions. Consequently, the SHAP importance associated with a given fingerprint bit should be interpreted as reflecting the influence of recurring structural patterns in a statistical context, rather than a direct mechanistic descriptor of interfacial polymerization dynamics. A repeated-split sensitivity analysis showed that removing the fingerprint block produced only minor changes in model performance (Table S7). Despite these interpretability limitations, the aggregated SHAP patterns remain valuable for guiding inverse design by highlighting statistically favorable or unfavorable descriptor patterns across the data set.

Overall, chemical features exhibit relatively lower SHAP values compared to the ion and membrane properties, suggesting a more modest impact on model predictions. Specifically, features such as f_1180 and f_1424 are associated with reduced salt rejection, while f_588, f_133, and f_322 correlate with lower water permeability, indicating these features may be unfavorable for high-performance membrane design. Conversely, f_1656 contributes positively to salt rejection, and f_360, f_1348, and f_361 enhance water permeability. These findings support the use of descriptor-level information as one component of candidate screening, alongside formulation and process variables. To screen potential monomers, we compared Tanimoto similarity between candidate monomers and reference structures derived from aggregated fingerprint patterns. These aggregated patterns served as illustrative screening references for ranking existing, commercially available monomers rather than as proposed molecular structures.^44^ For ML-Bayesian optimization, only commercially available monomers were considered. Monomers with a similarity score greater than 0.1 were shortlisted for high-performance membrane design.

### Designing Candidate Membranes Using ML-Bayesian Optimization

In interfacial polymerization, membrane fabrication conditions such as the types and concentrations of amine and acyl chloride monomers, as well as heat curing parameters, must be carefully selected. Given the vast design space, relying solely on trial-and-error approaches is impractical for developing high-performance membranes. To address this challenge, we combined ML with Bayesian optimization to prioritize candidate membrane designs toward targeted performance outcomes. The optimization process is driven by a custom loss function defined as |B/A| + |predicted water permeability—target water permeability|.

This function directs the search toward fabrication conditions that minimize deviations from the desired performance metrics. To apply the ML-Bayesian optimization framework, we first generated random combinations of input variables representing membrane fabrication conditions. These were fed into the trained ML model to predict corresponding salt rejection and water permeability values. The predicted outputs were then used to compute the loss, which guided the selection of candidate fabrication conditions. Based on this feedback, Bayesian optimization iteratively explores the design space, proposing new conditions likely to yield lower loss values, thus progressively guiding the process toward experimentally testable membrane designs.

### Experimental Evaluation of Optimized Membrane Design

To evaluate the AI-assisted membrane design framework, we selected membrane fabrication conditions to achieve a range of target water permeability values (4, 6, 8, 10, and 12 LMH/bar) while maintaining high salt rejections, as shown in Table 1. To simplify the design space, we fixed the solvent and acyl chloride components as hexane and TMC, respectively. The ranges of numerical input features were determined based on the training data set. For the 6 and 12 LMH/bar targets, two sets of fabrication conditions were presented. One set included polyethylenimine (PEI) as an amine component, but membranes synthesized under these conditions did not meet both performance metrics. Therefore, alternative non-PEI conditions were selected for experimental evaluation. The PEI-containing outcomes are discussed below as applicability-domain boundary cases.

Most selected amine compounds shared a common structural feature: the presence of two amine groups within or attached to a benzene, piperazine, or cyclohexane ring. This configuration facilitates cross-linking and polymerization during the IP process. Among these input variables, the molar concentration ratio of amine and acyl chloride monomers emerged as one of the most influential factors in the SHAP analysis, as shown in Figure 3. In general, higher target water permeability was associated with lower molar ratios. However, the relatively high ratio observed for the membrane targeting 10 LMH/bar deviated from this trend. Among the remaining optimized fabrication conditions, heat curing time was the only variable that showed a consistent pattern, generally decreasing from 20 to 5 min as the target permeability increased.

Based on the selected fabrication conditions, membranes were synthesized and tested for water permeability and salt rejection, as summarized in Table 2. When PEI was used as the amine source for the IP, a significant decline in either water permeability or salt rejection was observed. For example, the membrane targeting a water permeability of 6 achieved a high salt rejection of 98.3%, but the water permeability was limited to 1.5 LMH/bar. In another case targeting 12 LMH/bar, the membrane exhibited relatively high water permeability (14.7 LMH/bar); however, the salt rejection dropped to 59.2%, which is substantially below the target.

**Table 2:** Performance Results of Selected Fabricated Membranes Used for Experimental Validation

Target perm.	Water perm. (LMH/bar)	Salt rej. (%)	PEI as amine monomer
4	3.5 ± 0.4	99.4 ± 0.2	No
6	1.5 ± 0.1	98.3 ± 0.3	Yes
6	6.0 ± 0.1	96.4 ± 1.0	No
8	8.2 ± 0.4	98.2 ± 1.0	No
10	12.4 ± 1.0	95.2 ± 2.8	No
12	14.7 ± 2.5	59.2 ± 7.4	Yes
12	Active layer was not formed	Active layer was not formed	No

These PEI-containing membrane results are intentionally reported as applicability-domain boundary cases rather than as successful optimization outcomes. Their behavior may partly reflect limitations of the current monomer-level descriptor representation for polymeric amines such as PEI. Unlike amine monomers such as m-phenylenediamine (MPD) or piperazine (PIP), PEI is a polymer whose physicochemical properties depend on the number of repeating units and molecular weight, which are not fully captured when PEI is represented only by its repeating unit. Separately, limited training data at high water permeability may reduce prediction reliability. As illustrated in Figure S2A, most training data are concentrated between 2 and 10 LMH/bar, with relatively few samples near 12 LMH/bar. This places the 12 LMH/bar target in a data-sparse region, where predictions are supported by fewer nearby observations and are therefore less reliable.

It should be noted that the experimental evaluation presented here focuses on NF performance targeting a standard sulfate salt (Na_2_SO_4_) under fixed operating conditions, rather than demonstrating ion-specific separation across multiple salts or mixed-ion systems. In this context, Na_2_SO_4_ serves as a practical and widely adopted benchmark for evaluating NF performance, given that it is more readily rejected due to strong electrostatic interactions. As such, the present findings primarily reflect the combined contributions of size exclusion and charge-based rejection, rather than fully capturing ion-specific transport behavior. A more comprehensive assessment using salts with different valence characteristics (e.g., NaCl, MgCl_2_) would further elucidate these effects and extend the workflow to broader ion-specific separations. Nevertheless, because the data available for other salts were limited and variable, they were not sufficiently robust for statistically meaningful interpretation or reliable integration into the current ML framework. This limitation aligns with the data availability constraints discussed earlier and underscores the importance of balanced data sets in ML-guided membrane design. Within this scope, the role of ionic descriptors in this study is to support an inverse-design workflow informed by literature trends, rather than to experimentally resolve subtle ion-specific effects, which remain important directions for future work.

### Membrane Characterization

To characterize the experimentally evaluated non-PEI membranes, membranes with permeabilities of 3.5, 6.0, 8.2, and 12.4 LMH/bar were systematically analyzed to investigate their structural and physicochemical properties. FESEM analyses were used to examine the surface and cross-sectional morphologies of the membranes. As shown in Figure 4, clear differences were observed in the surface morphologies, whereas the cross-sectional images indicate that the active layer thicknesses are comparable across all membranes. The 3.5 LMH/bar membrane exhibited a finer and more compact nodular surface, whereas the 6.0 and 8.2 LMH/bar membranes showed more pronounced and rougher surface features. The 12.4 LMH/bar membrane displayed relatively coarser and less densely packed nodular domains, suggesting a more open surface structure. This interpretation is consistent with prior NF literature, which shows that variations in IP can significantly influence the nodular surface structure, roughness, and compactness of the PA layer.^69–71^ Consequently, these structural changes modulate water transport resistance and hence membrane permeability.

**Figure 4:**
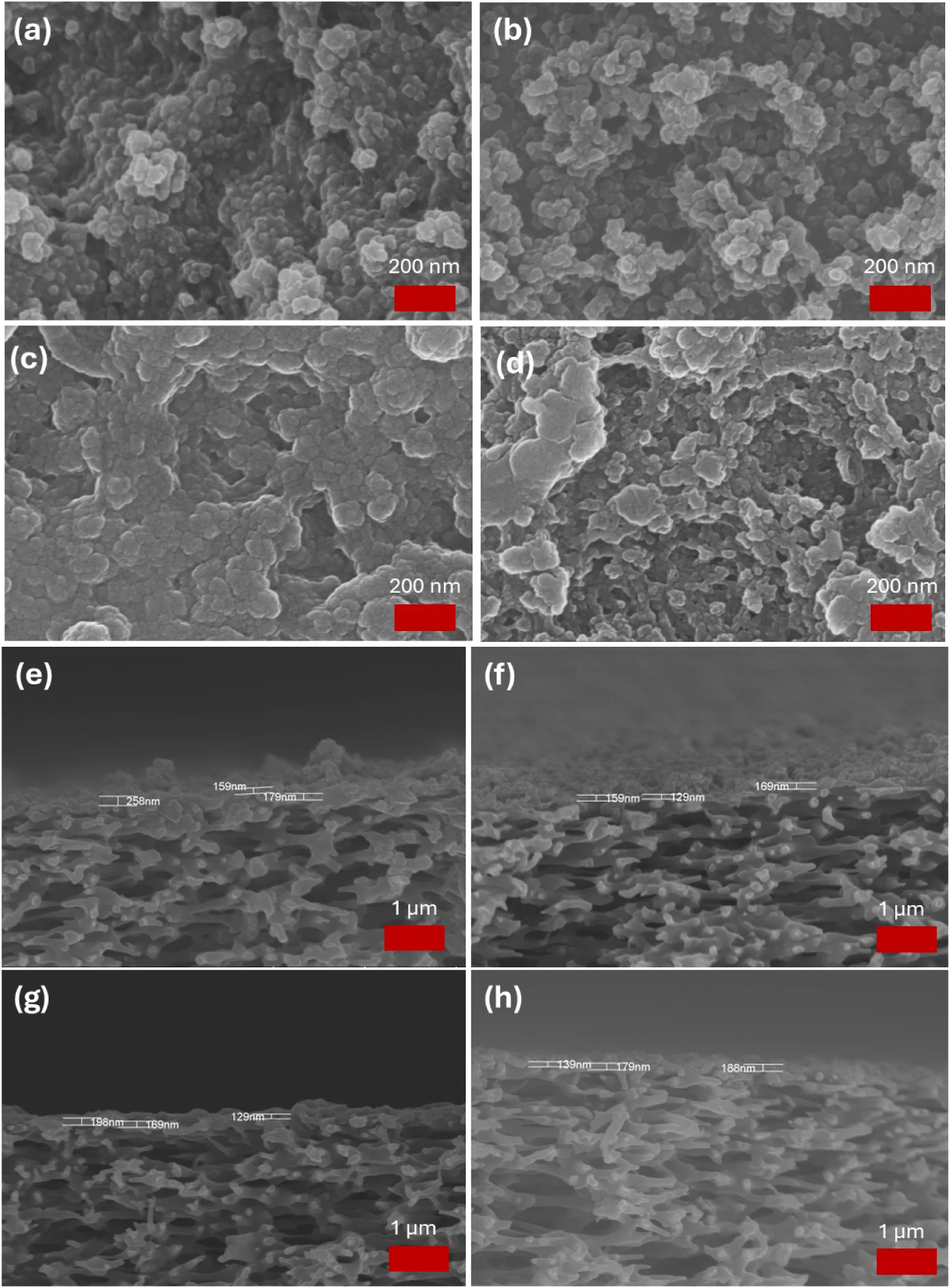
Surface (a–d) and cross-sectional FESEM images (e–h) of membranes with permeabilities of (a, e) 3.5 LMH/bar, (b, f) 6.0 LMH/bar, (c, g) 8.2 LMH/bar, and (d, h) 12.4 LMH/bar.

In contrast, cross-sectional observations did not show substantial differences in active layer thickness, indicating that the interfacial polymerization process maintained similar film thickness across the membranes. This consistency in active layer thickness suggests that the fabrication conditions were well controlled, minimizing thickness-induced variations in transport behavior. Because the cross-sectional images demonstrate comparable active-layer thicknesses, the observed differences in separation performance are more reasonably attributed to differences in effective pore size and surface charge. These factors govern water transport and solute rejection through size exclusion and electrostatic interactions, consistent with the established roles of size sieving and charge regulation in NF membranes.^72^ These results provide physical context for the measured permeability differences by linking transport behavior to effective pore structure and surface charge.

The separation characteristics of the membranes were quantitatively assessed through analysis of MWCO, mean pore size, pore size distribution, and surface charge (Figure 5 and Table 3). As illustrated in Figure 5, the MWCO values exhibit a systematic increase as a function of membrane permeability. The MWCO increased from 199 Da for the 3.5 LMH/bar membrane to 357 Da for the 12.4 LMH/bar membrane, indicating a progressive transition toward a less restrictive selective layer. This trend confirms that all fabricated membranes fall within the NF regime (typically 200–1000 Da), with the 4 LMH/bar membrane representing a tight NF membrane and the 12.4 LMH/bar membrane approaching the upper range of loose NF membranes.^73^ The mean pore size follows a consistent trend, increasing from 0.25 to 0.31 nm with increasing permeability. Although the absolute change in pore size appears small, such variations are highly significant at the NF scale, where even subnanometer differences can strongly influence solute transport. In parallel, the pore size distribution slightly broadens with increasing permeability, particularly for the 12.4 LMH/bar membrane (σ_p_ = 1.330), indicating increased structural heterogeneity and the presence of larger transport pathways. These structural changes directly explain the observed permeability trend. As pore sizes increase and the distribution broadens, the hydraulic resistance of the selective layer decreases, leading to higher water permeability. This behavior is consistent with classical transport models for NF membranes, where water flux is strongly dependent on effective pore size and pore connectivity.^74^

**Table 3:** Physicochemical Properties of the Fabricated Membranes

Membrane permeability (LMH/bar)	Rejection (%)	MWCO (Da)	Mean pore size (r̅) (nm)	σ_p_	Zeta potential (mV, pH = 7.0)
3.5	99.4	199	0.25	1.199	–21.8
6.0	96.4	255	0.27	1.239	–3.2
8.2	98.2	275	0.29	1.234	–12.7
12.4	95.2	357	0.31	1.330	–3.6

**Figure 5:**
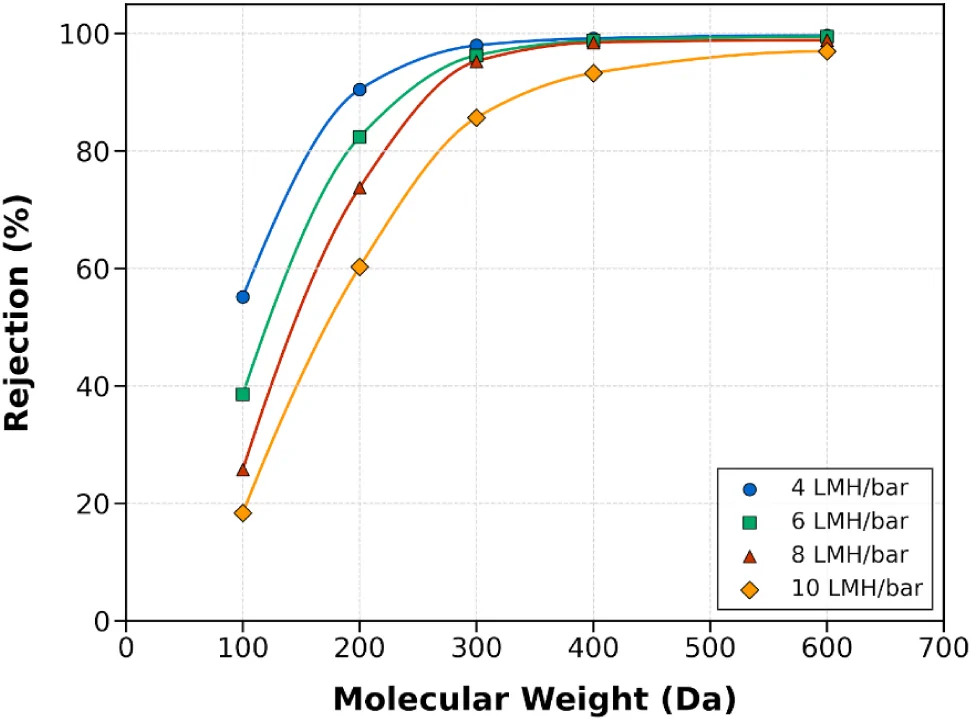
Rejections of neutral solutes with different molecular weights for MWCO determination.

Importantly, the rejection behavior cannot be explained by pore size alone and is strongly influenced by surface charge effects. Zeta potential measurements reveal that the 3.5 LMH/bar membrane has a highly negative surface charge (−21.8 mV), whereas membranes with higher permeability exhibit significantly less negative values (−3.15 to −12.7 mV). This reduction in surface charge density diminishes electrostatic repulsion toward SO_4_^2–^, which is highly sensitive to charge-based exclusion. This observation underscores that rejection is controlled by the combined mechanisms of size exclusion and Donnan exclusion.^75^ The highly negative surface charge of the 3.5 LMH/bar membrane enhances electrostatic repulsion, resulting in near-complete rejection. In contrast, the 6.0 and 12.4 LMH/bar membranes exhibit much less negative surface charges (−3.15 and −3.62 mV, respectively), resulting in reduced electrostatic exclusion and contributing to the observed decrease in rejection. Interestingly, the 8.2 LMH/bar membrane showed a more negative zeta potential (−12.7 mV) compared to the 6.0 LMH/bar membrane. This accounts for the relatively higher rejection (98.2% vs 96.4%) despite its larger pore size. The surface charge efficiently offsets the increased pore size, consistent with the synergistic steric hindrance and electrostatic interactions.^76^ These measurements provide physical context for the experimentally evaluated membranes and are consistent with the permeability-selectivity trends targeted by the MLAID workflow.

## Environmental Implications

In this study, we presented a data-driven framework that integrates ML coupled with BO to accelerate candidate prioritization and experimental planning for membrane design. MLAID enables systematic screening of monomer candidates and fabrication parameters, reducing the experimental search burden while targeting permeability and selectivity. We experimentally evaluated selected monomer candidates under prioritized fabrication conditions across a range of targeted permeability values. The results show how candidate prioritization can be coupled to experimental evaluation, with boundary cases feeding back into descriptor and data set requirements for future design cycles. This direction is relevant to future water treatment and resource recovery applications.

Broader applications, including emerging-contaminant removal and chlorine-resistant membranes, are natural next targets for this workflow as target-specific data sets and descriptors become available. By organizing existing evidence and prioritizing experiments, this strategy can support the search for membrane materials that reduce reliance on chemical-intensive treatment processes. It may also help address a key limitation of conventional polyamide membranes—their vulnerability to oxidative degradation by chlorine. Extending the workflow to chlorine-resistant materials would require target-specific data sets, descriptors, and experimental validation, but the same ML/BO backbone could guide the development of more robust, fit-for-purpose membranes.

Looking ahead, this research lays the groundwork for designing membranes with tailored selectivity for multicomponent separations, such as binary, ternary, or more complex mixtures. This capacity supports both contaminant removal and recovery of high-value resources such as solvents, nutrients, and minerals. Future iterations of this framework can incorporate additional performance indicators, including antifouling properties and thermal, chemical, and mechanical stability, to guide the development of durable membranes optimized for diverse industrial applications. The current ML model was initially developed as a stand-alone application without requiring high-performance computers, enabling NF/RO membrane design by prioritizing promising monomer and fabrication-condition combinations for targeted separation performance. Beyond the present study, MLAID could be developed into a cloud-based platform that supports broader access and the integration of models for nanofiltration, reverse osmosis, gas separation, and other membrane processes. This expansion will require larger and more diverse data sets, uncertainty-aware optimization strategies, and further experimental validation. In this context, the current study provides an initial workflow template for linking modeling, optimization, and experimental evaluation in membrane science. By integrating domain-specific knowledge with automated optimization, MLAID could evolve into a versatile platform supporting material design across multiple sectors.

## Supplementary Material



## Data Availability

The curated data sets and model-training and Bayesian-optimization notebooks are openly available at https://github.com/Nohyeong/ML-BO_membrane_design.

## Notes

The curated input-variable list, hyperparameter ranges, residual and monomer-level diagnostic figures, SHAP dependence analyses, and supplementary repeated-split, same-input-condition, simple-baseline, and fingerprint-block sensitivity diagnostics (Tables S3–S7) (PDF).
