# Supplementary material for: Learning the Path to Ion Separation: A Proof-of-Concept for Machine Learning-Assisted Inverse Design (MLAID) of Nanofiltration Membranes
Source: Environ Sci Technol. 2026 Sep 29;60(43):31006–19. doi: 10.1021/acs.est.5c15241 (PMC13738799; doi:10.1021/acs.est.5c15241)
Supplement: Supplementary file 1 [file es5c15241_si_001.pdf]

Supporting Information for

Learning the Path to Ion Separation: A Proof-of-Concept for Machine Learning-Assisted Inverse Design (MLAID) of Nanofiltration Membranes

Submitted to: Environmental Science & Technology

Nohyeong Jeong#, Elif Demirel#, Changyoon Jun and Yongsheng Chen*

School of Civil & Environmental Engineering, Georgia Institute of Technology, Atlanta, GA, 30332, United States

#These authors contributed equally to this work.

*Corresponding author: yongsheng.chen@ce.gatech.edu

Number of Pages: 10

Number of Figures: 10

Number of Tables: 7

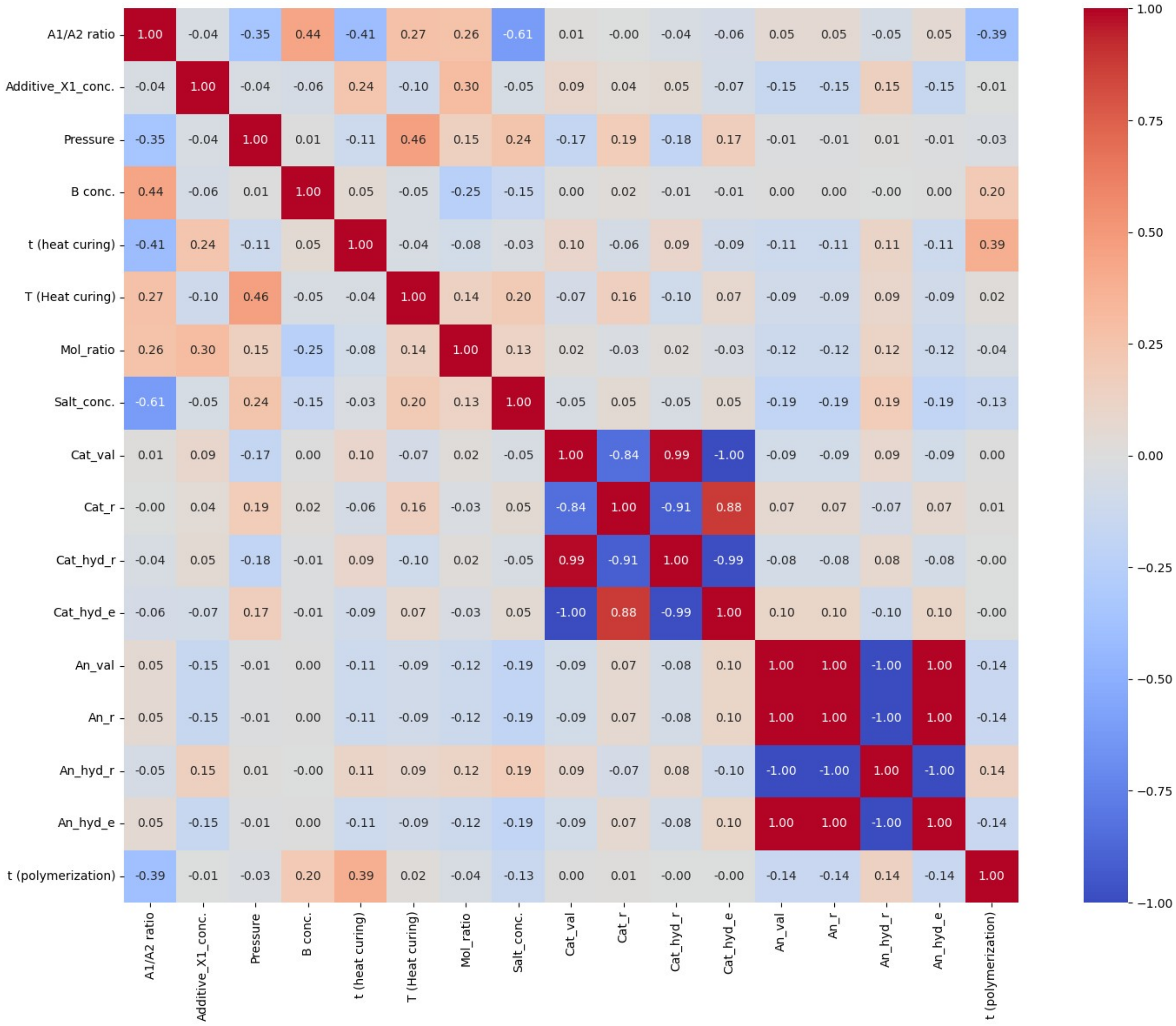

Figure S1. Correlation matrix of input variables in the training data.

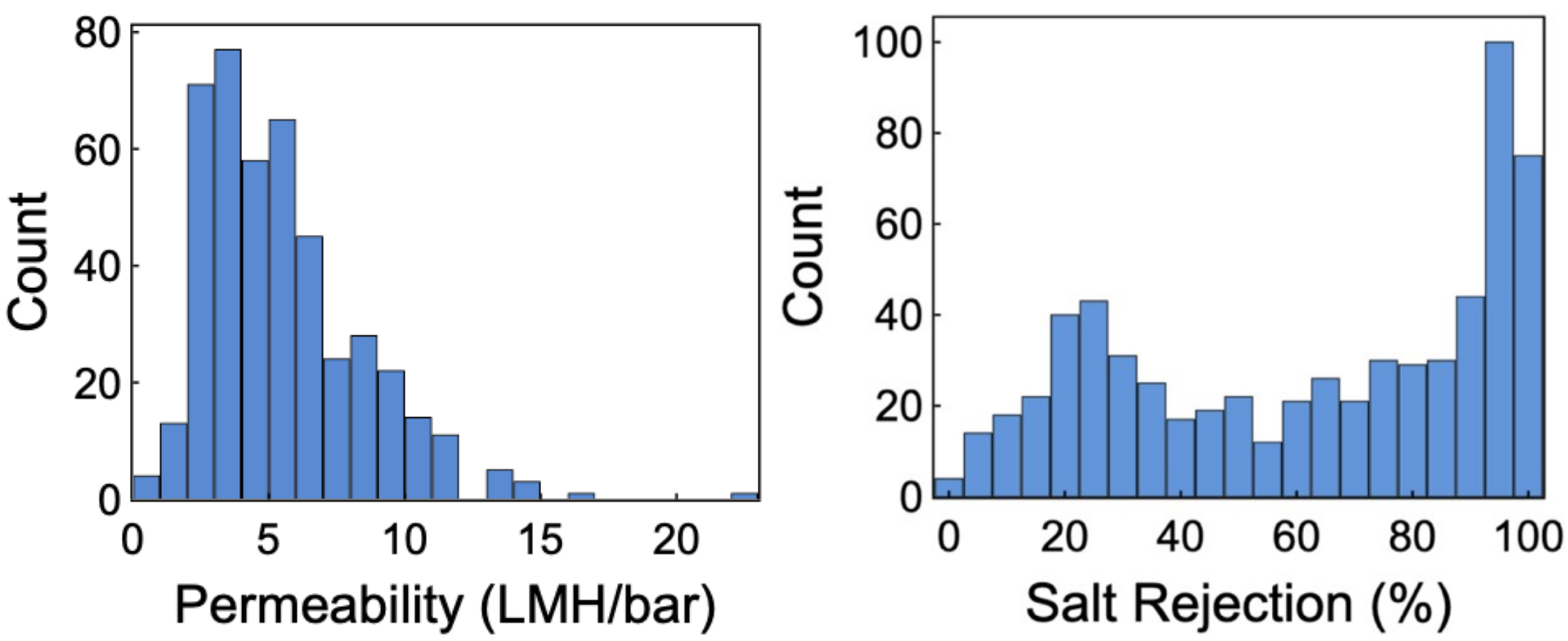

Figure S2. Histograms of permeability and salt rejection data in the dataset

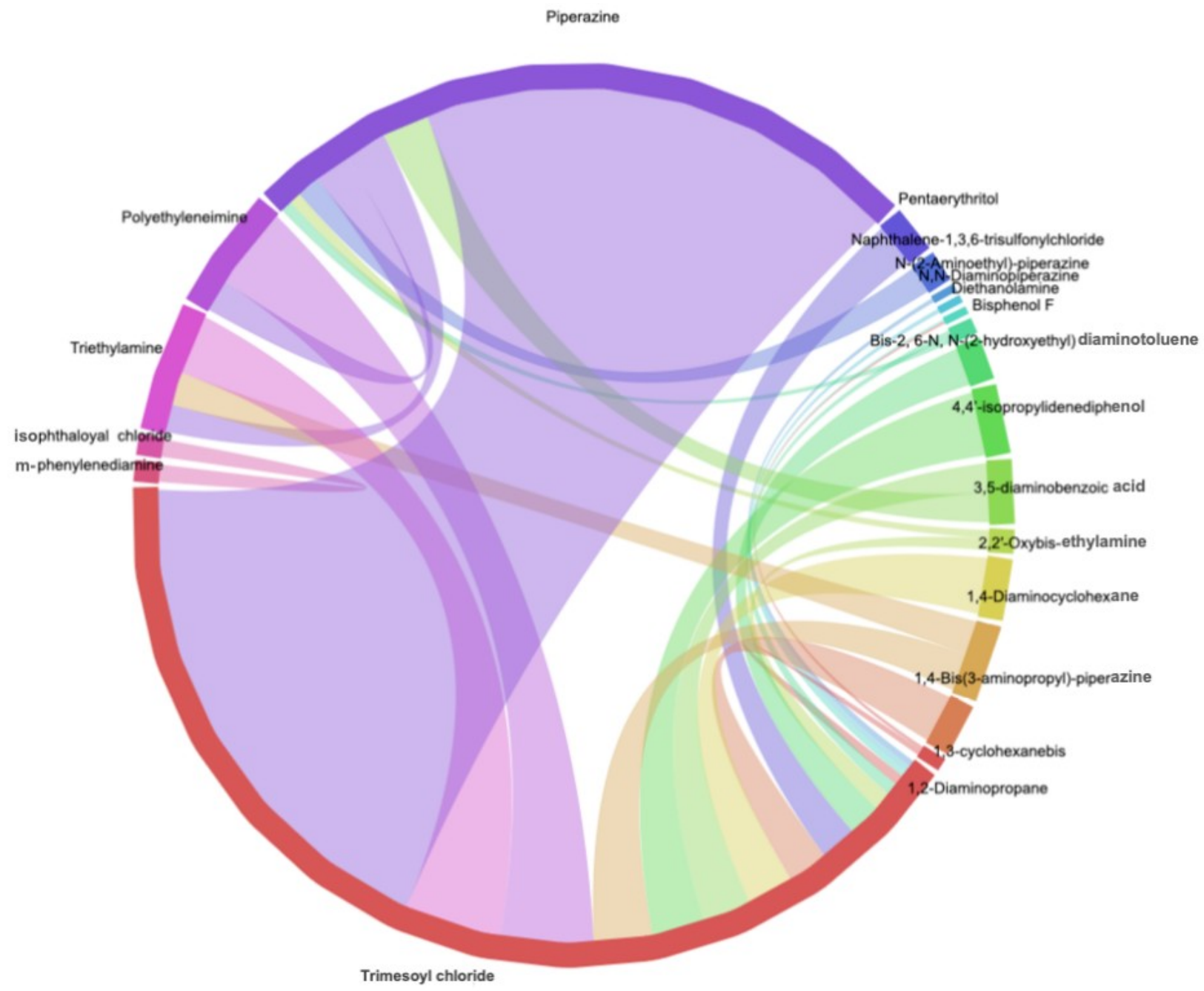

Figure S3. Chord diagram showing the relationships between monomers used in interfacial polymerization datasets for permeability prediction.

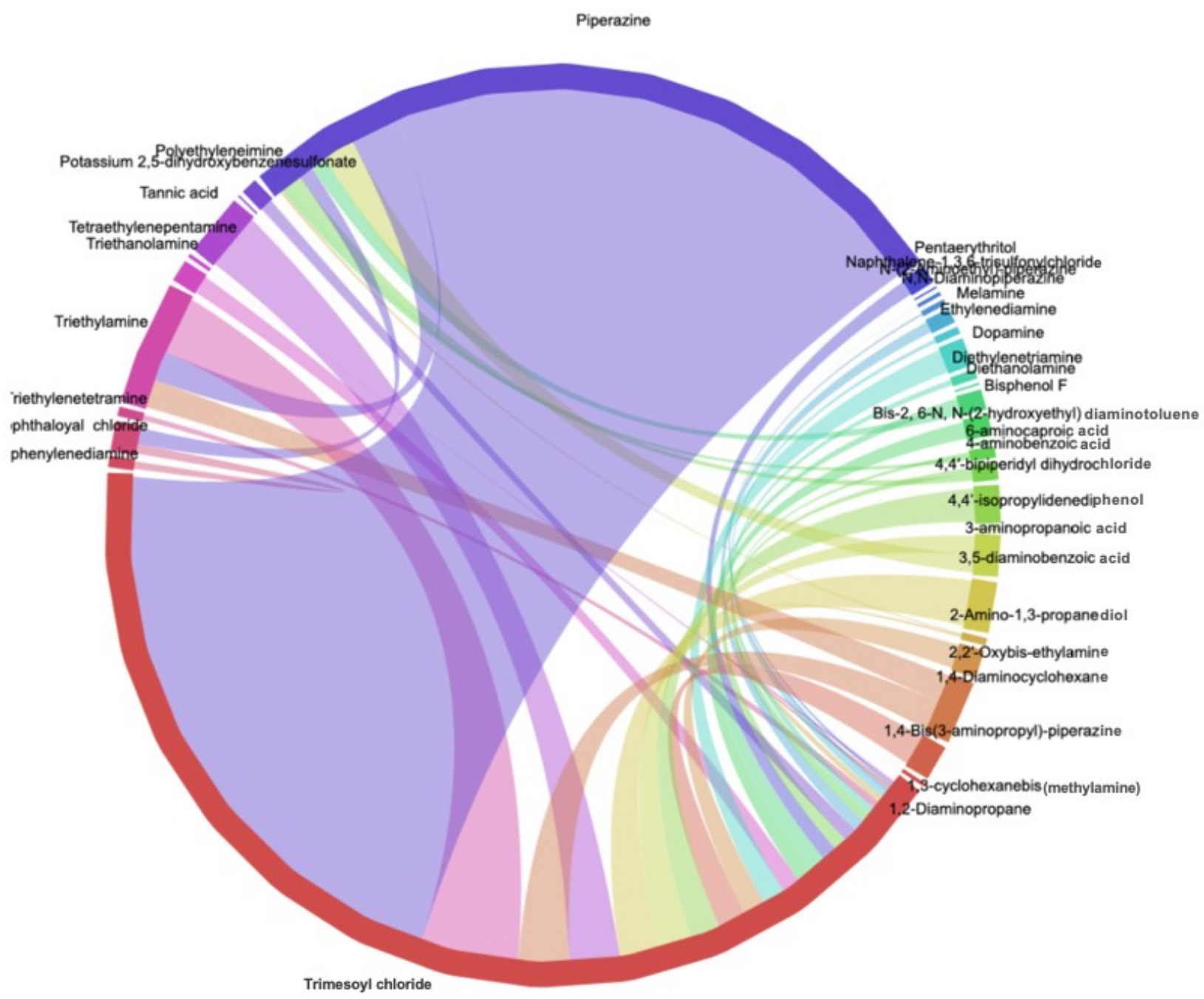

Figure S4. Chord diagram showing the relationships between monomers used in interfacial polymerization datasets.

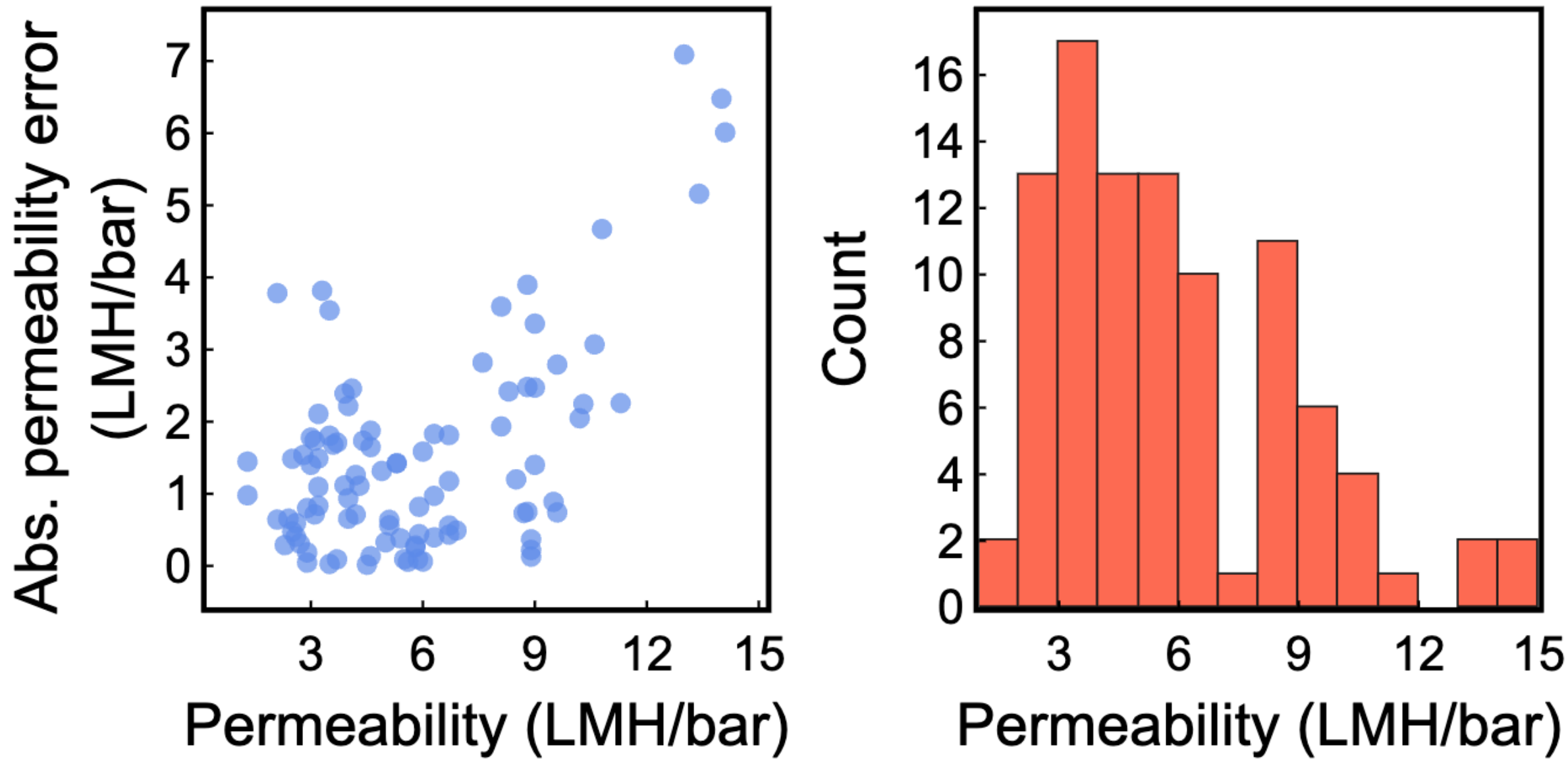

Figure S5. Residual analysis of model performance. (Left) Scatter plot of absolute permeability error as a function of permeability and (Right) histogram of permeability values in the dataset.

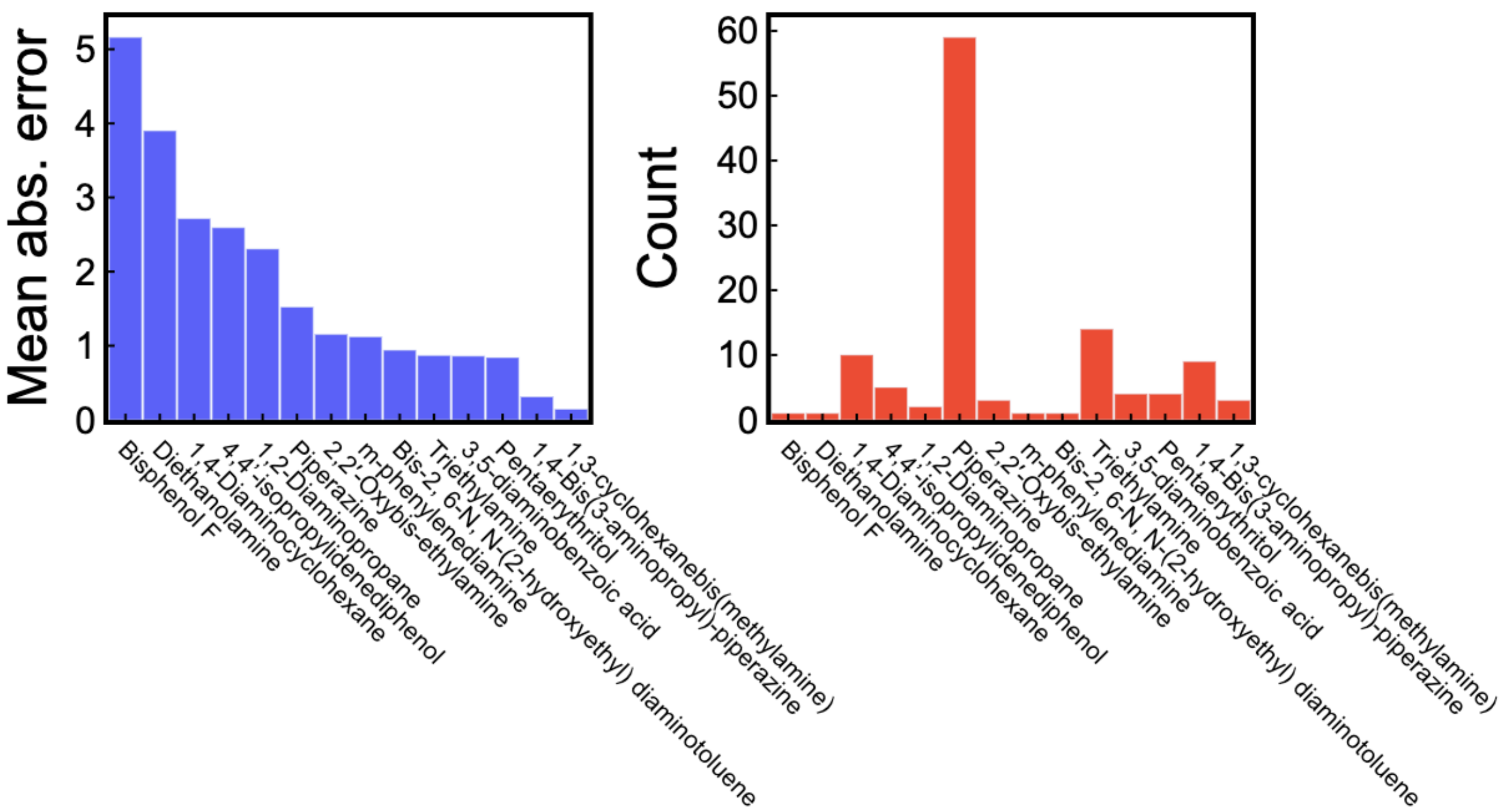

Figure S6. Mean absolute error of salt rejection prediction by monomer type. (Left) Mean absolute error of salt rejection predictions grouped by monomer type. (Right) Number of samples associated with each monomer type used in the model evaluation.

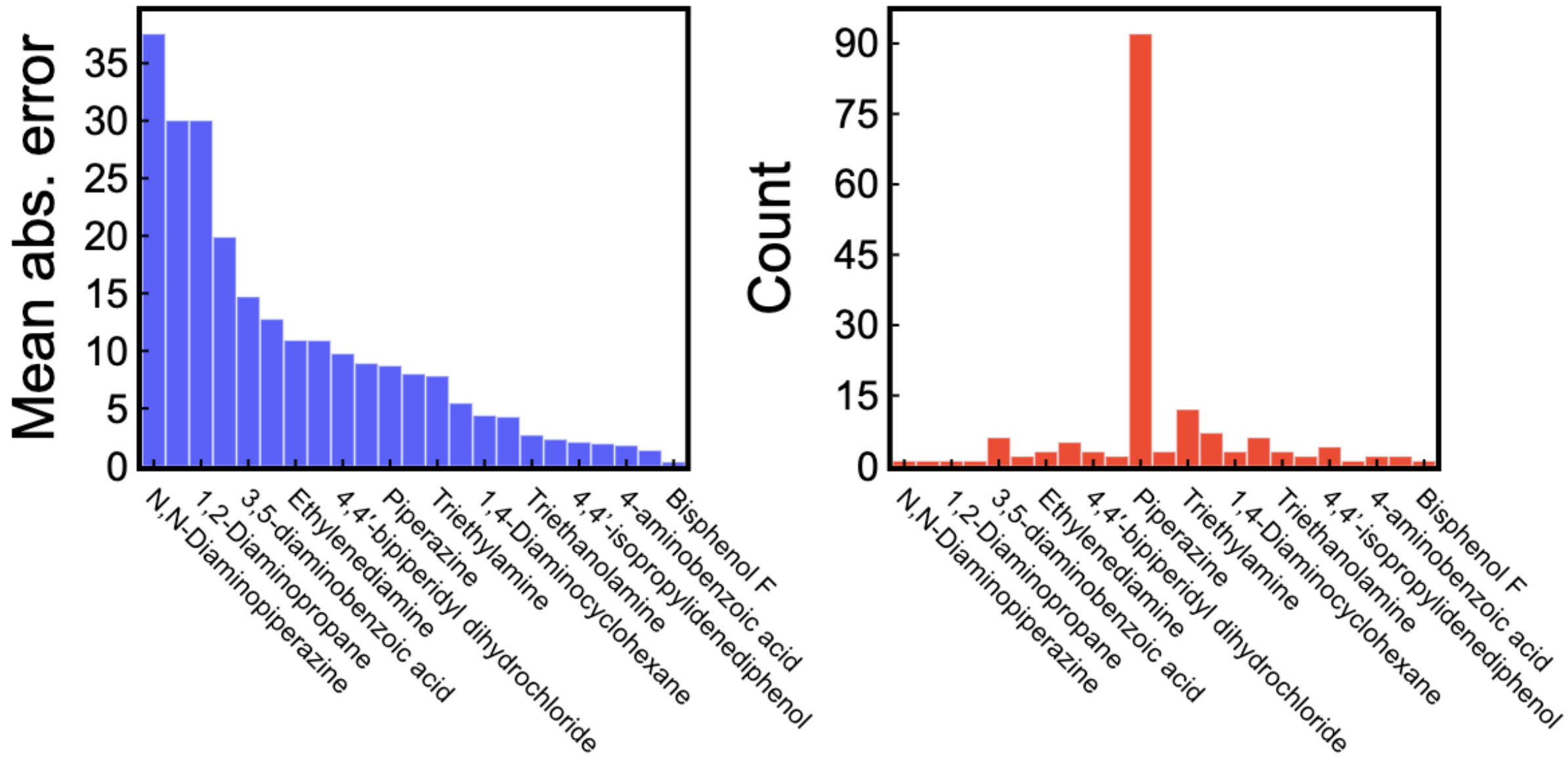

Figure S7. Mean absolute error of permeability prediction by monomer type. (Left) Mean absolute error of permeability predictions grouped by monomer type. (Right) Number of samples associated with each monomer type used in the model evaluation.

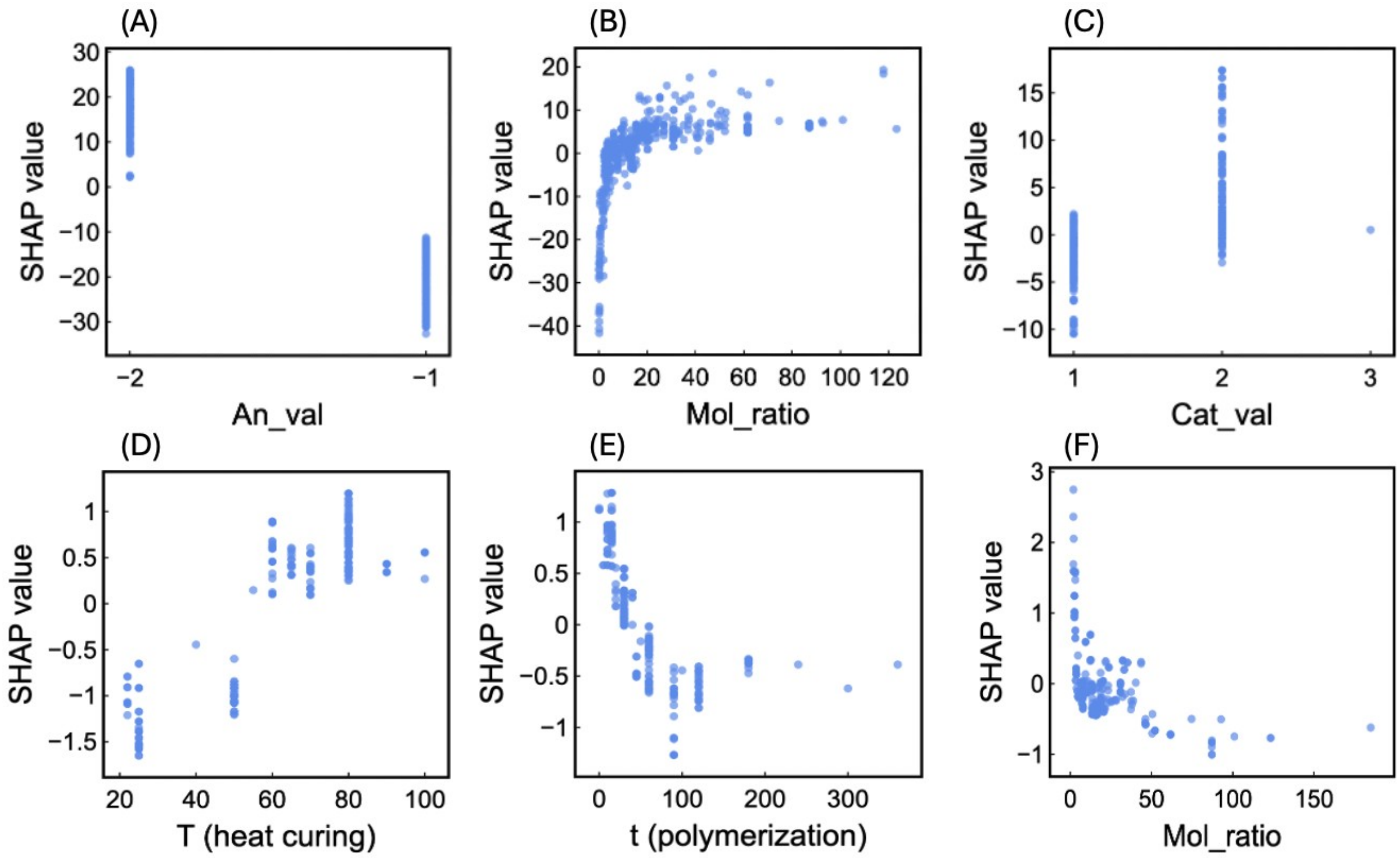

Figure S8. SHAP dependence plots for salt rejection and permeability models. (A-C) SHAP dependence plots for the salt rejection model, showing SHAP values as a function of anion valence, molar ratio of monomer A and B, and cation valence, respectively. (D-F) SHAP dependence plots for the permeability model, showing SHAP values as a function of heat curing temperature, polymerization time, and molar ratio of monomer A and B, respectively.

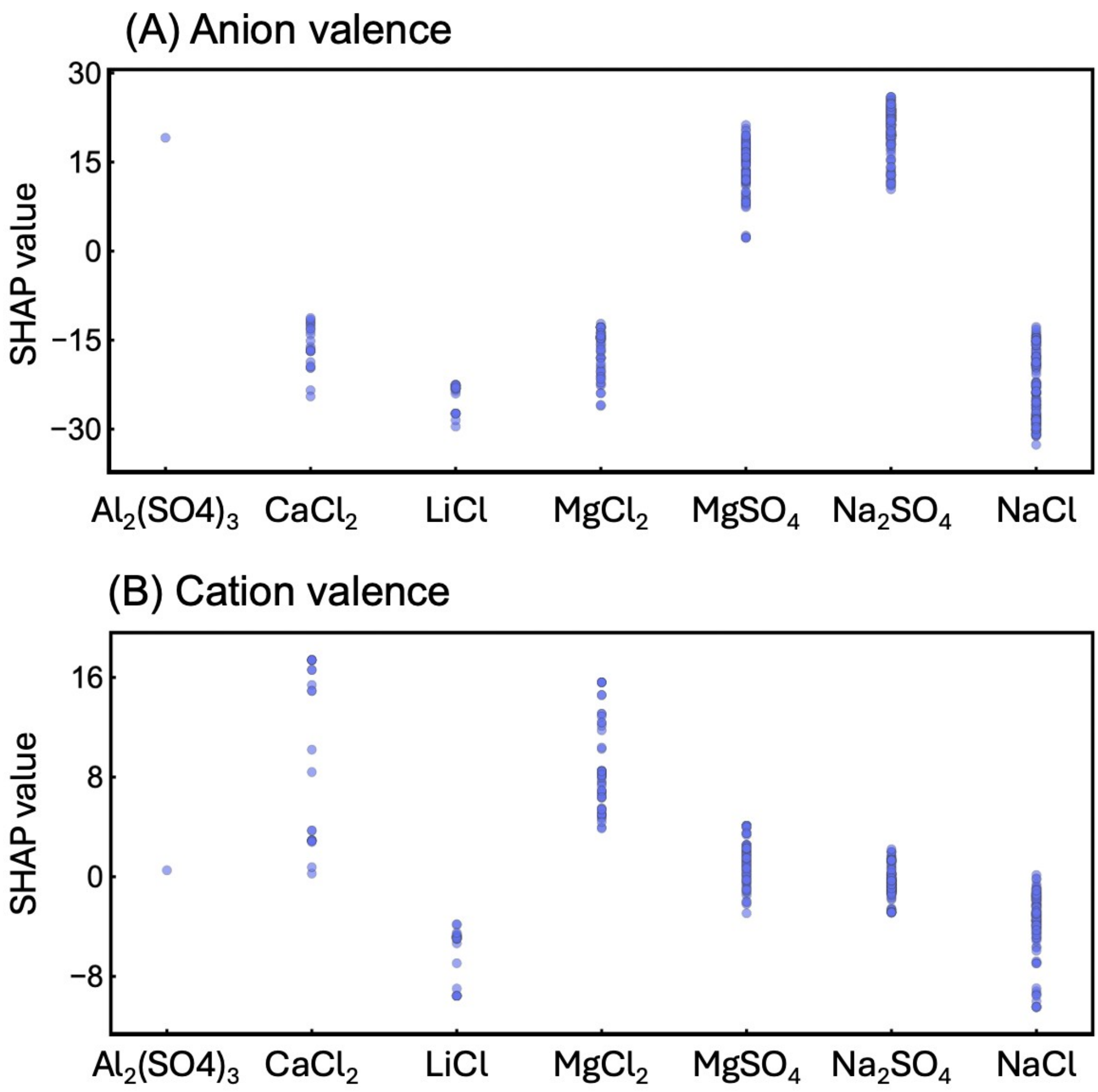

Figure S9. Comparison of SHAP values illustrating the contribution of (A) anion valence and (B) cation valence to the salt rejection predictions. Both variables were used in model training. Salt names are provided in the figure to facilitate interpretation of SHAP values.

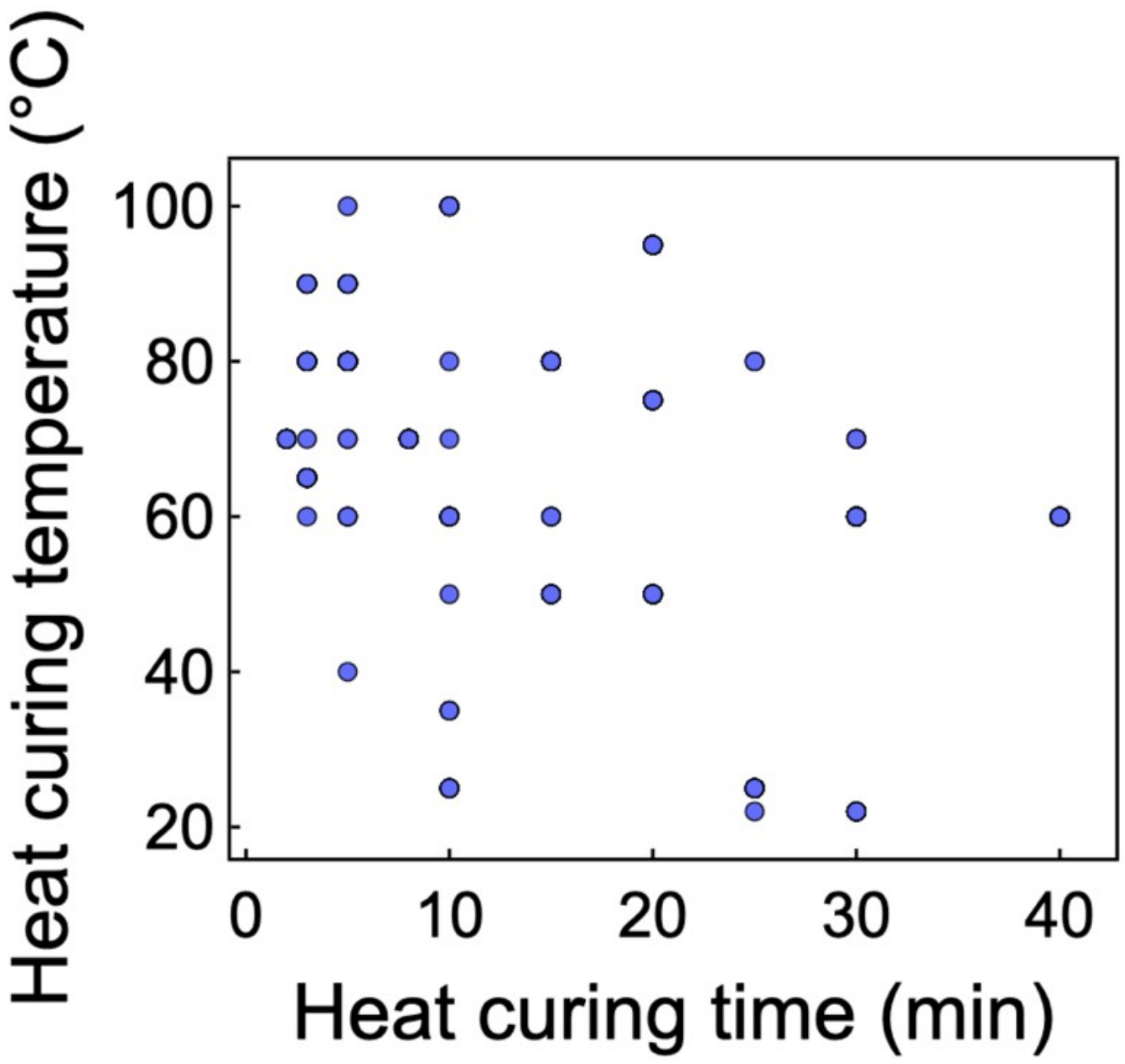

Figure S10. Relationship between heat-curing temperature and curing time in the permeability dataset.

Table S1. Complete model input-variable list and model-specific usage.

Design parameter	Abbreviation	Rej.	Perm.
Anion valence	An_val	√	√
Mol concentration ratio of amine and acyl chloride monomers	Mol conc. ratio	√	√
Cation valence	Cat_valence	√	√
The concentration of acyl chloride monomer	B conc.	√	—
Salt concentration	Salt_conc.	√	√
Cation radius	Cat_radius	√	√
Heat curing temperature	T (heat curing)	√	√
Polymerization time	t (polymerization)	√	√
The concentration of additive X1	Additive_X1_conc	√	√
Heat curing time	t (heat curing)	√	√
Hydrated radius of cation	Cat_hyd_radius	√	√
First additive (aqueous phase) in the dataset	Additive_X1_0	√	√
Second additive (aqueous phase) in the dataset	Additive_X1_1	√	√
Fourth additive (aqueous phase) in the dataset	Additive_X1_3	√	√
First organic solvent in the dataset	Org. solvent_0	√	√
Hydration energy of cation	Cat_hyd_energy	√	√
Anion radius	An_radius	√	√
Hydrated radius of anion	An_hyd_radius	√	√
Hydration energy of anion	An_hyd_energy	√	√

Design parameter	Abbreviation	Rej.	Perm.
A1/A2 monomer concentration ratio	A1/A2 ratio	√	√
Monomer B identity	Monomer B type	√	—
Transmembrane pressure	Pressure	√	—
Total concentration of amine monomers (A1 + A2)	monomer_tot_wt	—	√
Morgan fingerprint block for monomer structures	Morgan FP (4096 bits)	√	√

Note: A check mark indicates that the variable was included in the corresponding salt-rejection or water-permeability model. Rej. and Perm. denote the salt-rejection and water-permeability models, respectively. The Morgan fingerprint row summarizes the encoded fingerprint block rather than listing each bit separately.

Optimization method. Both hyperparameter tuning and the inverse-design candidate search were performed with the Tree-structured Parzen Estimator (TPE) algorithm implemented in the hyperopt library, a sequential model-based (Bayesian) optimization method. TPE does not fit a Gaussian-process surrogate; instead, it models the densities of better- and worse-performing trials and proposes candidates by their density ratio (which corresponds to maximizing Expected Improvement under these densities), using the hyperopt default settings (including 20 random start-up evaluations). For inverse design, the trained salt-rejection and water-permeability models were used as fixed surrogates within the scalar objective L = |B/A| + |predicted - target permeability|, and TPE searched the formulation/fabrication space (amine-monomer identities from a curated candidate list; A1/A2 ratio; monomer and additive concentrations; polymerization time; heat-curing time and temperature), while the acyl chloride monomer (TMC), organic solvent (hexane), transmembrane pressure (5 bar), and Na_2SO_4 ion descriptors were held fixed. The search used a fixed evaluation budget with no additional early-stopping criterion; the selected candidates are the lowest-loss formulations under this objective and were treated as experimentally testable hypotheses rather than unique global optima.

Table S2. The list of hyperparameters and their ranges

Hyperparameters	Range
max_delta_step	0-10
learning_rate	0.001-0.1
max_depth	1-10
min_child_weight	1-10
Subsample	0.5-1.0
reg_alpha	0-10
Gamma	0-10
reg_lambda	0-10
n_estimators	1-1000

Supplementary Model Diagnostics

Tables S3-S7 provide repeated-split, same-input-condition exclusion, baseline, and fingerprint-block sensitivity diagnostics within the curated dataset.

Table S3. Seed-level repeated random-row split diagnostics.

Task	Seed	R^2	RMSE	MAE
Salt rejection	0	0.82	13.40	8.25
Salt rejection	1	0.87	11.38	7.06
Salt rejection	2	0.83	13.51	8.15
Salt rejection	3	0.88	11.12	7.71
Salt rejection	4	0.86	11.37	7.34
Salt rejection	5	0.80	13.37	8.46
Salt rejection	6	0.85	12.02	7.41
Salt rejection	7	0.72	15.06	9.74
Salt rejection	8	0.83	12.23	8.28
Salt rejection	9	0.85	12.29	7.82
Water permeability	0	0.37	2.08	1.37
Water permeability	1	0.26*	2.58	1.35
Water permeability	2	0.61	1.84	1.25
Water permeability	3	0.56	1.99	1.30
Water permeability	4	0.58	1.76	1.23
Water permeability	5	0.50	1.82	1.31
Water permeability	6	0.59	1.76	1.16
Water permeability	7	0.57	1.93	1.20
Water permeability	8	0.18*	2.79	1.52
Water permeability	9	0.27	2.64	1.83

*Note: The split seed determines only the 80/20 assignment of observations; the underlying dataset and model settings are unchanged. For water permeability, the two lowest R2 values (seeds 1 and 8, marked with *) occurred when the single 22.5 LMH/bar observation was assigned to the test set.*

Table S4. Mean ± SD summary of repeated random-row split diagnostics.

Task	R^2	RMSE	MAE
Salt rejection	0.83 ± 0.05	12.58 ± 1.24	8.02 ± 0.76
Water permeability	0.45 ± 0.16<br>(0.51 ± 0.12)	2.12 ± 0.40	1.35 ± 0.20

Note: Values are reported as mean ± SD across the ten splits summarized in Table S3. For water permeability, the value in parentheses (0.51 ± 0.12) is the mean ± SD across the eight splits in which the 22.5 LMH/bar observation remained in the training set. The lower ten-split average therefore reflects sensitivity to this single high-permeability observation.

Table S5. Exact-duplicate input-condition exclusion diagnostic summary.

Task	R^2	RMSE	MAE
Salt rejection	0.84 ± 0.04	12.17 ± 1.52	7.96 ± 0.73
Water permeability	0.53 ± 0.14	2.02 ± 0.37	1.32 ± 0.15

Note: Exact duplicate input-condition rows were removed before repeated random-row splitting. Values are reported as mean ± SD across the seed sets summarized in Table S3.

Table S6. Comparison of XGBoost with a training-set-mean baseline on the held-out test splits used for Figure 2.

Model	R^2	RMSE	MAE
**Rejection (salt rejection, %)**			
Training-set mean	−0.003	32.63	29.65
XGBoost	0.87	11.95	7.51
**Permeability (LMH/bar)**			
Training-set mean	−0.004	2.99	2.42
XGBoost	0.63	1.82	1.22

Note: The training-set-mean predictor and XGBoost were evaluated on the same held-out 80/20 test splits used for Figure 2.

Table S7. Fingerprint-block sensitivity across 10 random train/test splits.

Target	ΔR^2	ΔRMSE
**Rejection**	-0.014 ± 0.007	0.558 ± 0.232
**Permeability**	-0.011 ± 0.025	0.022 ± 0.050

Note: Values are reported as mean ± SD across 10 random 80/20 train/test splits (seeds 0–9). Δ values are calculated as performance after removal of the Morgan fingerprint block minus full-model performance.
